# Quantifying production rates and size fractions of parrotfish‐derived sediment: A key functional role on Maldivian coral reefs

**DOI:** 10.1002/ece3.8306

**Published:** 2021-11-03

**Authors:** Robert T. Yarlett, Chris T. Perry, Rod W. Wilson

**Affiliations:** ^1^ Geography College of Life and Environmental Sciences University of Exeter Exeter UK; ^2^ Biosciences College of Life and Environmental Sciences University of Exeter Exeter UK

**Keywords:** carbonate production, coral reefs, functional roles, parrotfish, sediment production, sediment reworking

## Abstract

Coral reef fish perform numerous important functional roles on coral reefs. Of these, carbonate sediment production, as a by‐product of parrotfish feeding, is especially important for contributing to reef framework construction and reef‐associated landform development. However, only limited data exist on: (i) how production rates vary among reef habitats as a function of parrotfish assemblages, (ii) the relative importance of sediment produced from eroded, reworked, and endogenous sources, or (iii) the size fractions of sediment generated by different parrotfish species and size classes. These parameters influence not only overall reef‐derived sediment supply, but also influence the transport potential and depositional fate of this sedimentary material. Here, we show that parrotfish sediment production varies significantly between reef‐platform habitats on an atoll‐margin Maldivian reef. Highest rates of production (over 0.8 kg m^−2^ year^−1^) were calculated in three of the eight platform habitats; a rubble‐dominated zone, an *Acropora* spp. dominated zone, and a patch reef zone. Habitat spatial extent and differences in associated parrotfish assemblages strongly influenced the total quantities of sediment generated within each habitat. Nearly half of total parrotfish sediment production occurred in the rubble habitat, which comprised only 8% of the total platform area. Over 90% of this sedimentary material originated from eroded reef framework as opposed to being reworked existing or endogenously produced sediment, and comprised predominantly coral sands (predominantly 125–1000 µm in diameter). This is comparable to the dominant sand types and size fractions found on Maldivian reef islands. By contrast, nearly half of the sediment egested by parrotfish in the *Acropora* spp. dominated and patch reef habitats resulted from reworked existing sediments. These differences between habitats are a result of the different parrotfish assemblages supported. Endogenous carbonate production was found to be insignificant compared to the quantity of eroded and reworked material. Our findings have important implications for identifying key habitats and species which act as major sources of sediment for reef‐island systems.

## Graphical abstract

Our findings show that parrotfish sediment production varies between reef habitat types as a function of the species assemblages and fish sizes supported. The origins of this material (i.e., bioerosion of reef substrate, sediment reworking, or endogenous production) also varies between habitat types because of the species and sizes of parrotfish present. In addition, we show that all species studied produce sediment size fractions suitable for shoreline maintenance.

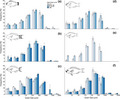

## 1 INTRODUCTION

Reef sediment production is a critical process that can influence the formation and maintenance of coral reef habitats, such as sandy lagoons and seagrass meadows as well as reef‐associated landforms, such as beaches and reef islands (Hutchings, 1986; Kench & Cowell, 2000; Perry et al., 2015). Reef sediment production can result from physical (mechanical disturbance by waves and storms) and chemical (ooid formation), as well as biological (scraping, excavating, etching, boring, and endogenous production by reef organisms) processes. This includes sediment derived directly post‐mortem from skeletal taxa such as mollusks and foraminifera, and that generated from the erosion of the reef substrate during feeding by taxa such as fish and urchins (Glynn, 1997; Perry et al., 2012; Perry & Hepburn, 2008). While difficult to quantify the relative importance of each process, biological sediment production is known to be a significant source of reef sediments (Bellwood, 1995b; Glynn, 1997; Perry et al., 2015; Scoffin et al., 1980). Sediment production as a by‐product of parrotfish feeding has been identified as being especially important in some regions (Morgan & Kench, 2016; Perry et al., 2015). This is one of a suite of important fish functions linked to feeding (other examples include grazing, browsing, and bioerosion) that can directly contribute to physically and ecologically shaping reef environments.

The two main parrotfish functional groups associated with substrate modification and sediment production are the “scrapers” (that scrape the reef substrate) and “excavators” (that take larger and deeper bites from reef substrate) (Bellwood, 1995; Bellwood & Choat, 1990; Nanami, 2016; Ong & Holland, 2010). Using their oral jaws, parrotfish erode reef framework (Alwany et al., 2009; Bellwood, 1995a; Bellwood et al., 2003; Bruggemann et al., 1996; Morgan & Kench, 2016a) whilst feeding predominantly on dead coral substrates (Afeworki et al., 2011; Bellwood, 1995a; Bruggemann et al., 1994), although live coral can be a substantial (up to 50%) feeding substrata for some species (Bonaldo et al., 2014). In doing this, parrotfish are thought to be exploiting a range of dietary resources collectively known as microscopic photoautotrophs (Clements et al., 2017; Nicholson & Clements, 2020, 2021). In addition, through a process known as sediment reworking, parrotfish also consume, process, and egest loose sediments that have settled on these substrates or have been retained in the Epilithic Algal Matrix (EAM) (Bellwood, 1996; Bruggemann et al., 1996; Scoffin et al., 1980; Tebbett et al., 2017). While feeding, parrotfish erode and ingest reef framework and loose sediment along with organic matter, which is then broken down by modified gill arch elements known as the pharyngeal mill (Bellwood & Choat, 1990; Carr et al., 2006), processed in the gut, and egested back into the environment as sediment (Bellwood, 1995b, 1996; Morgan & Kench, 2016a). Sediment production rates for individual parrotfish are estimated to range from <3 to over 5000 kg year^−1^, assuming that bioerosion equals the rate of new sediment production (Bellwood, 1996; Bellwood et al., 2003). In the central Indian Ocean, parrotfish have been estimated to account for over 85% of biological sediment production on some Maldivian coral reefs (Perry et al., 2015, 2017). Post production, the hydrodynamic and depositional fate of this material is influenced by factors, such as grain size, density, and shape (Braithwaite, 1973; Kench, 1997; Kench & McLean, 1996). While these parameters generally exert a non‐uniform influence on carbonate sediment hydrodynamic behavior (Braithwaite, 1973), grain size is a fundamental property influencing sediment transport and deposition (Blott & Pye, 2001).

Most marine bony fish are also known to produce calcium carbonate endogenously as a by‐product of osmoregulation, primarily to remove excess calcium ions from the body and prevent renal stone formation (Perry, Kench, et al., 2011; Walsh et al., 1991; Wilson et al., 1996, 2009; Wilson & Grosell, 2003). The carbonate products of this are then egested into the environment in mucus‐coated pellets and may contribute fine (silt grade) carbonates (low to high Mg calcite, aragonite, and amorphous carbonates) to benthic sediments (Perry, Salter, et al., 2011; Salter et al., 2012). While endogenous carbonate production has now been investigated in a number of fish families, including one species of Caribbean parrotfish (Salter et al., 2012), the quantitative importance of this process in excavating and scraping parrotfish in the Indo‐Pacific remains unknown.

The total quantity of sediment produced by parrotfish, and the relative importance of new sediment generated by bioerosion (assuming sediment production equals erosion rate) and reworked existing sediment, varies with species and body size (Lange et al., 2020). The same may also be true for new endogenously produced sediment. Hoey and Bellwood (2008) examined variability in parrotfish functional roles (including sediment production and reworking) on inner, mid, and outer‐shelf environments on the Great Barrier Reef, but there has been little further work examining how overall rates of parrotfish sediment production and reworking vary between habitat types in other coral reef ecosystems. In addition, while some studies have examined sediment grain sizes produced for a range of parrotfish species (including Gygi, 1975; Bellwood, 1996; Hoey & Bellwood, 2008 and Morgan & Kench, 2016a), we have little understanding of the grain types (origin) of sediment produced by parrotfish, or how sedimentary characteristics change across different size classes of parrotfish. To advance our understanding of these areas, we: (1) investigated total rates of parrotfish sediment production across eight different habitat types, as a function of parrotfish species and body size, at Vavvaru Island, a small reef platform on Lhaviyani Atoll in the central Maldives, (2) estimated relative contributions of new sediment (derived from bioerosion and endogenous production) and reworked benthic sediments to total sediment production, and (3) determined the sedimentary characteristics (grain size and origin) of the material produced by a range of representative species and size classes of parrotfish in the Maldives. This was based upon existing published data on parrotfish presence and calculated bioerosion rates at Vavvaru (Yarlett et al., 2018, 2020), combined with new data on sediment reworking rates, endogenous carbonate production, and grain size and origin analysis.

## 2 MATERIALS AND METHODS

### 2.1 Study site

**FIGURE 1:**
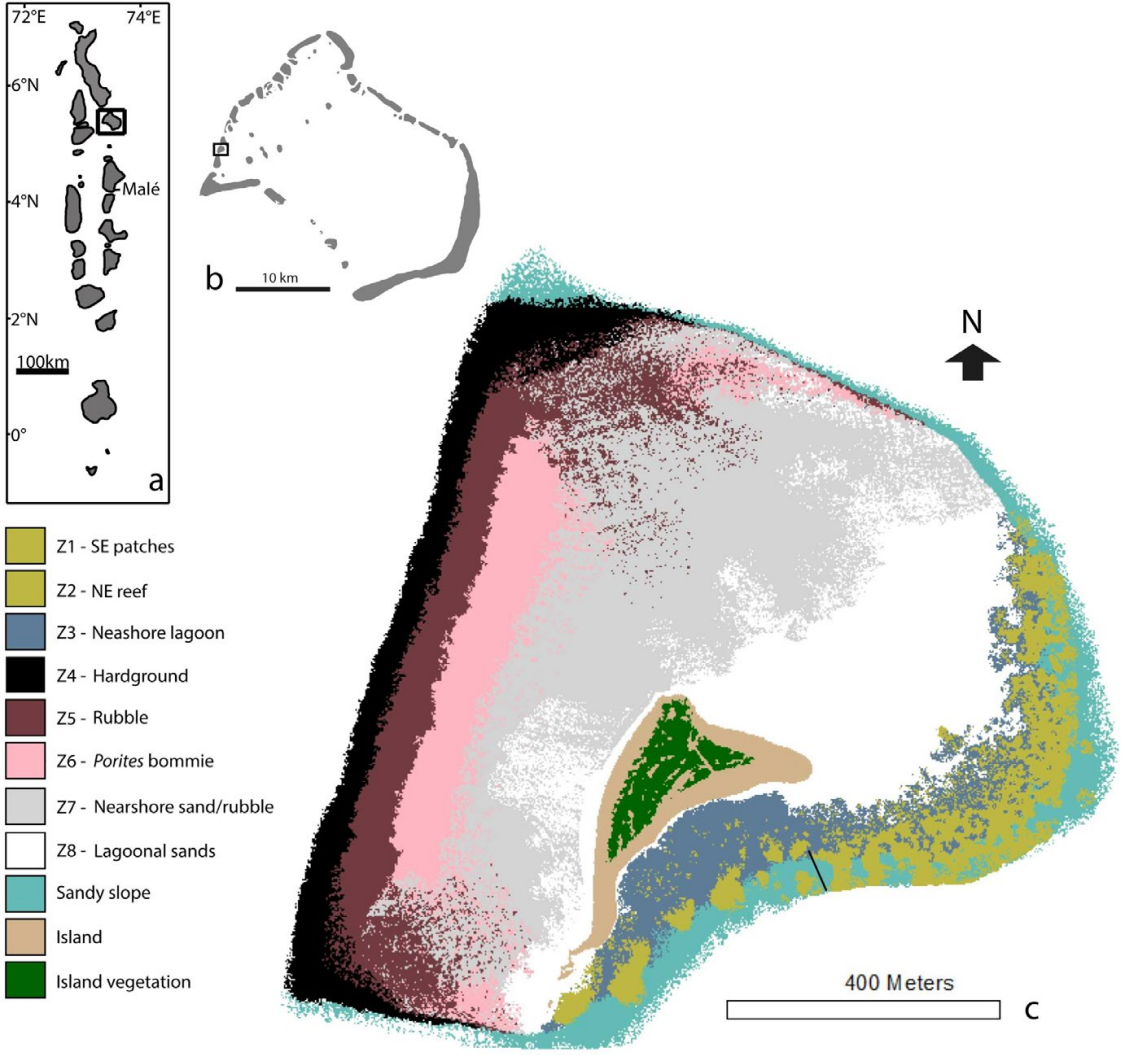
(a) Location of Lhaviyani Atoll in the Maldives. (b) Location of Vavvaru on Lhaviyani Atoll. (c) Habitat map of Vavvaru produced from Quickbird imagery of western Lhaviyani Atoll taken on 09/07/2008 (provided by DigitalGlobe Foundation; http://www.digitalglobefoundation.org/) and ground validated points. See Perry et al. (2017) for original publication and position of ground points

Field data were collected in early 2015 from an atoll edge reef platform, Vavvaru, Lhaviyani Atoll, in the northern‐central Maldives (N 5°25′5.0″; E 073°21′14.0″). Additional parrotfish fecal samples (methods described below) were collected in early 2016 from reefs on Gaafu Dhaalu Atoll, Southern Maldives to complement Vavvaru samples. The reef platform at Vavvaru comprised eight distinct marine habitats, as described in Perry et al. (2017) (Figure 1). Work on endogenous carbonate production by parrotfish was conducted on conspecifics at Lizard Island, Australia at the Lizard Island Research Station because of the excellent laboratory and aquarium facilities available, which were not available at sites in the Maldives.

### 2.2 Total parrotfish sediment production

Total parrotfish sediment production was estimated using an extended version of the calculation presented in Bellwood (1996), where total sediment production (TSP) was calculated from direct estimates of parrotfish bioerosion (or “primary erosion” – PE), loose sediment intake (or “reworked sediment” – RS), and the addition of endogenous production (EP). This was estimated at both the individual parrotfish and habitat scale (i.e., the sum of total parrotfish erosion, sediment reworking, and endogenous production in each habitat).

$$\text{TSP}\;\left(\text{kg}\;m^{‐2}\;{\text{year}}^{‐1}\right)=\text{PE}\;\left(\text{kg}\;m^{‐2}\;{\text{year}}^{‐1}\right)+\text{RS}\;\left(\text{kg}\;m^{‐2}\;{\text{year}}^{‐1}\right)+\text{EP}\;\left(\text{kg}\;m^{‐2}\;{\text{year}}^{‐1}\right).$$

Details of how each of these parameters were calculated, using both new and existing published data, are detailed in following sections of the methods. Endogenous carbonate production was assumed to be zero. This is because, despite evidence of some endogenous carbonate production, our results (see below) show that this process is likely insignificant compared to the quantity of sediment generated from bioerosion and sediment reworking in scraping and excavating parrotfish.

### 2.3 New sediment production from parrotfish bioerosion

Sediment production was assumed to match direct estimates of bioerosion rate (as assumed in other studies, such as Bellwood, 1996) because there is currently no clear evidence for dissolution of carbonates within the gut and there have also been no attempts to quantify the amount of material that parrotfish remove from the substrate, but do not ingest. Parrotfish also have tightly spaced gill rakers, which minimizes loss of sediment through the gills (Clements et al., 2017), so it is unlikely that any significant quantities of sediment were lost via “winnowing” (sensu Weller et al., 2016) as, for example, observed in some sediment feeding surgeonfish. Data on annual bioerosion rates for range of representative parrotfish species (and body sizes) at Vavvaru, and total annual habitat bioerosion rates were extracted from Yarlett et al. (2018), Yarlett et al. (2020). Habitat scale rates were estimated using observations of parrotfish occurrence and residence time within a survey area over a set time period (observed using remote underwater videos) and bioerosion estimates for a range of representative species at Vavvaru (*Chlorurus strongylocephalus*, *Chlorurus sordidus*, *Scarus rubroviolaceus*, *Scarus frenatus*, *Scarus niger*, and *Scarus psittacus*), originally presented in Yarlett et al. (2018). For species in which there were no data available, the same assumptions of bioerosion rates were made as detailed in Yarlett et al. (2020) and are summarized in Table S1.

### 2.4 Parrotfish sediment reworking – estimating rates

It was assumed that all parrotfish bites on reef substrate ingested loose sediment retained within the Epilithic Algal Matrix (EAM), as assumed in Bellwood (1996). The grazing scars observed in all habitats in the present study typically “cleaned” the area of the bite down to the underlying substrate, so it was assumed that all sediment retained within the bite area was ingested (the method for quantifying benthic sediment load is detailed below). To estimate sediment reworking rates, the surface area of substrate grazed per bite (published in Yarlett et al., 2020) by different size classes of each parrotfish species was multiplied by the quantity of sediment found in that unit area of substrate for each habitat, as assumed in Bellwood (1996). Individual sediment reworking rates were then calculated as follows:

$$\text{Sediment reworked per individual}\;\left(g\;{\text{min}}^{‐1}\right)=\text{Bites per minute}\;\left(\text{bpm}\right)\times \text{Sediment ingested per bite}\;\left(g\right)$$

Annual sediment reworking rates for each size class of each species in each habitat were then estimated using data on parrotfish observations at Vavvaru over a specified duration within a survey area (using remote underwater videos), extracted from Yarlett et al. (2020). Daily variation in bite rates was accounted for by calculating morning (sunrise −11:30), midday (11:30–14:00), and afternoon (14:00–sunset) average bite rates as described in Yarlett et al. (2020). Firstly, sediment reworking rates for each size class of each species during each survey were estimated using the following equation:

$$\text{TSR}\;{\text{(kg survey area}}^{‐1}\;{\text{survey duration}}^{‐1})=I\times \text{RT}\;\left(s\right)\times \text{RS}\;\left({\text{kg ind}}^{‐1}\;s^{‐1}\right)$$

where TSR is the Total Sediment Reworking rate (for each size class of each species), *I* is the number of individuals observed, and RT is the residence time.

These values were then converted to sediment reworking rates per m^2^ by dividing by the survey area, and then to an Annual Reworking Rate (ARR) per m^2^ by scaling to the length of the feeding day (11 h; Yarlett et al., 2018) and multiplying by 365 days^−1^. This was repeated for all 15 replicate video surveys in each habitat before finding an Average Annual Reworking Rate (AARR) for each size class of each species.

Finally, total sediment reworking rates for each habitat were estimated using the following equation:

$$\text{TAHR}\;\left({\text{kg habitat area}}^{‐1}\;{\text{year}}^{‐1}\right)=\sum \text{AARR}\;\left({\text{kg m}}^{‐2}\;{\text{yr}}^{‐1}\right)\times \text{habitat area}\;\left(m^{2}\right)$$

where TAHR is the total annual habitat reworking. Habitat areas were derived from the habitat map in Figure 1 as described in Perry et al. (2017).

### 2.5 Parrotfish sediment reworking – sediment load within the epilithic algal matrix (EAM)

Three loose substrate samples (~50 cm^−2^), on which parrotfish had been observed to feed, were randomly collected from each habitat where parrotfish were found (Hardground – Z4, Rubble – Z5, *Porites* bommie – Z6, NE reef – Z2, SE patch reefs – Z1, and the eastern Nearshore Lagoon – Z3). No rubble samples were collected from the western Nearshore sand/rubble – Z7 or Lagoonal sands – Z8 habitats because no parrotfish were observed there, so it was assumed sediment reworking by parrotfish was minimal or absent in those habitats. Each rubble sample was retrieved from the reef and carefully placed in a zip lock bag to be transported to the laboratory. The exposed surface of the rubble samples (i.e., the surface that parrotfish were able to feed on) was carefully rinsed and scrubbed using a wire brush to remove loose sediment and collected in a beaker, taking care not to dislodge sediment from other surfaces. The collected sediment was rinsed in distilled water to remove salts, soaked in 5% sodium hypochlorite solution (bleach) to neutralize organics, and rinsed a further two times in distilled water to remove bleach residues before being dried and weighed. During each cleaning step, the sediment was left long enough (~3 h) to fully settle out before decanting the supernatant. This ensured that all sediment was retained but reduced unnecessary soaking time, which may have increased the likelihood of dissolution. The surface area of each substrate sample was measured by wrapping foil around the exposed surface, which was then removed, laid flat, and photographed next to a ruler used for scale. The surface area of the foil, which corresponds to that of the rubble substrate, was then measured using the software image J. This method was chosen over collecting sediment in a quadrat or hoop area using underwater vacuums due to the topographic heterogeneity of the substrate, and so to avoid the risk of overestimating sediment load in the samples.

### 2.6 Endogenous carbonate production

To investigate whether parrotfish produce endogenous carbonates, an additional set of experiments was carried out on conspecifics collected on reefs around Lizard Island, Australia. Parrotfish (3 × *Chlorurus spilurus* and 15 × *S. psittacus*) were collected using barrier nets and transported to aquaria in aerated seawater transport containers. Individuals were then grouped by species and size and kept in aerated aquaria with running seawater pumped from local shallow waters. These aquaria were filtered to 1 µm to minimize external sediment or organic matter inputs and thereby preventing fish from ingesting sediment material during sampling. Temperature, pH, and salinity in the aquaria were regularly monitored and ranged between 29 and 32°C, a pH of 8.00–8.23, and a salinity of 34. False floors (mesh raised ~4 cm from the bottom of the tank) were used to allow fecal pellets to sink out of reach of the fish. Fish were left unfed for 2 days to allow egestion of any food ingested prior to capture and adjust to aquarium conditions. The aquaria were then thoroughly cleaned before sample collection. Any carbonates produced from this point were assumed to be produced endogenously and were collected within 24 h of egestion using Pasteur pipettes. These carbonates were then rinsed with distilled water to remove salts and soaked in 5% sodium hypochlorite for ~20 min to remove organic components. Two additional rinse steps were applied to ensure removal of salt and bleach residues. The cleaned samples were oven‐dried at 40°C and packaged for transport. Once sample collection was completed, the fish were transported in aerated containers by boat and released at the same site that they were caught.

### 2.7 Grain size and origin analysis – parrotfish fecal sample collection

Fecal samples for sedimentary analysis were collected in the field (Maldives field sites) from initial and terminal phase *C. sordidus*, *C. strongylocephalus*, *S. niger*, *S. frenatus*, *S. psittacus*, and *S. rubroviolaceus* in the following size classes: <15 cm, 16–30 cm, 31–45 cm, and >46 cm (*n* = >5 per size class per species). Individuals of target study species were followed until egestion was observed. Egested fecal pellets were collected using a large bulb pipette and transferred to individual 15‐ml falcon tubes. Samples were only collected when target fish egested close to the seafloor to minimize any sediment dispersal, and when egested material was deposited on an accessible substrate with minimal potential for contamination by benthic sediments. Egestion events where sediment was observed to disperse were ignored. This approach was used successfully in Morgan and Kench (2016a) and was chosen to avoid harvesting over 150 parrotfish for gut content analysis, although it may result in an underestimate of fine‐grained sediments as detailed in the discussion. Samples were transported to the laboratory, where they were left to settle out before decanting the seawater and rinsing in distilled water to remove salts. The samples were then bleached (5% sodium hypochlorite) for ~15 min to neutralize organics and transferred to a vacuum filter chamber with 0.4 µm Whatman cellulose nitrate filter. Samples were then rinsed thoroughly in ~50 ml of distilled water. The filter with retained sediments was then removed from the chamber and dried. Sediments were then poured off the filter paper into a sample tube, and any sediments retained on the filter were gently scraped off using a blunt pair of tweezers to ensure retention of fines.

### 2.8 Grain size and origin analysis – sediment size fractions and grain identification

The grain size distribution of the parrotfish fecal sediments was measured using laser diffraction. Five replicate samples of each size class of each target parrotfish species were analyzed using a Malvern mastersizer 2000, which measures the equivalent spherical volume of each grain. Five technical replicates of each of these samples were collected to account for any variation due to irregular grain shapes. Grain size classes are reported following the Udden–Wentworth nomenclature.

The proportion of different grain types in these sediment samples was also examined using a Scanning Electron Microscope (SEM). Subsamples of dried parrotfish fecal sediments were prepared by mounting onto aluminum SEM stubs using a double‐sided adhesive and coated with 20 nm of Gold/Palladium before being imaged under the SEM (with a working distance of ~16 mm, an operating voltage of 10 kv, and spotsize of 30). A series of images (usually incorporating 50–100 grains) with no overlap were taken systematically across the stub until at least 300 grains from the sample were imaged. Each grain was identified, using the images taken, into one of the following broad categories: Coral, Crustose Coralline Algae, *Halimeda* spp., Mollusca, Foraminifera and where accurate ID was not possible, Unidentified. Images of comparable sediment samples from the literature were used to support visual identification of grains (studies included Adjas et al., 1990; Perry, 2000; Scoffin & Tudhope, 1985). A minimum of 300 grains were identified per stub as considered representative of the composition (Cheetham et al., 2008). A thorough search for endogenous carbonate grain morphologies was also carried out at high magnification (×4000) allowing clear view of grains <2 µm in diameter in each sample.

## 3 RESULTS

### 3.1 Habitat‐scale rates of parrotfish sediment production and reworking

**FIGURE 2:**
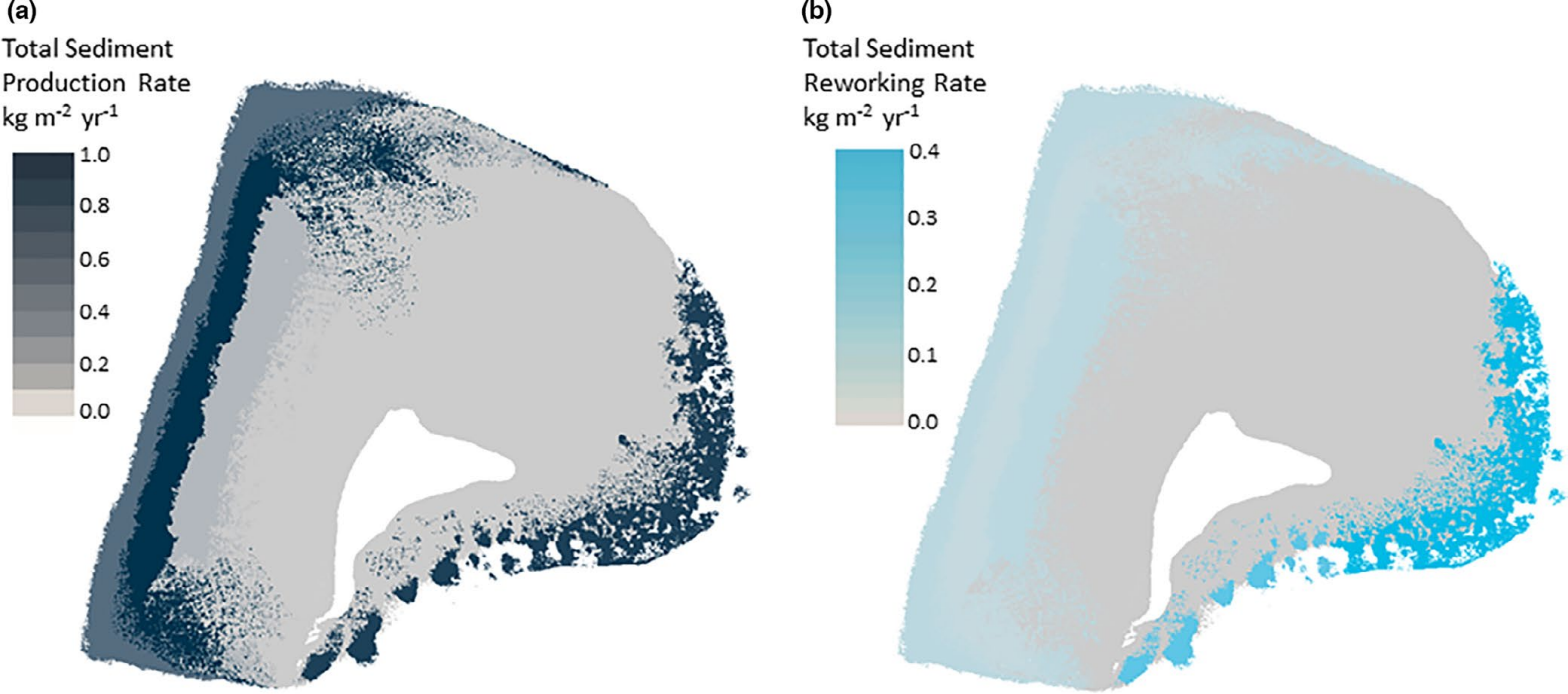
Choropleth maps showing (a) Inter‐habitat variability in total sediment production by parrotfish over the Vavvaru platform and (b) Inter‐habitat variability in sediment reworking rates over the Vavvaru platform

Overall parrotfish sediment production rates were highest in the Rubble – Z5, NE reef – Z2, and SE patch reef – Z1 habitats, all of which produced over 0.8 kg sediment m^−2^ year^−1^ (Figure 2A). However, a significant proportion (>34%) of sediment in the eastern reef habitats was reworked existing sediment (Figure 2B, Table 1). By comparison, nearly all (~92%) of the sediment generated by parrotfish in the Rubble habitat – Z5 resulted from bioerosion of reef substrate.

**TABLE 1:** Rates and total annual quantities of parrotfish sediment production in Vavvaru reef habitats, and the contribution of reworked sediment

	Z1	Z2	Z3	Z4	Z5	Z6
Total sediment production (kg year^−1^)	12,760 ± 1174	44,814 ± 4908	1083 ± 185	40,067 ± 7647	87,775 ± 11151	13,921 ± 1020
% Contribution to total platform parrotfish sediment production	6%	22%	1%	20%	44%	7%
Reworked sediment (kg year^−1^)	4347 ± 486	20,941 ± 3539	804 ± 114	5924 ± 448	7272 ± 808	7570 ± 647
Total sediment production rate (kg m^−2^ year^−1^)	0.88 ± 0.08	0.87 ± 0.10	0.02 ± 0.00	0.58 ± 0.11	0.91 ± 0.12	0.17 ± 0.01
Sediment reworking rate (kg m^−2^ year^−1^)	0.30 ± 0.03	0.41 ± 0.07	0.01 ± 0.00	0.09 ± 0.01	0.08 ± 0.01	0.09 ± 0.01
% of Total sediment reworked	34%	47%	74%	15%	8%	54%

Sediment reworking rates were considerably higher in the eastern reef habitats (NE reef – Z2: 0.41 ± 0.07 kg m^−2^ year^−1^; SE patch reef – Z1: 0.30 ± 0.03 kg m^−2^ year^−1^) compared to the western Hardground – Z4, Rubble – Z5, and *Porites* bommie – Z6 habitats (0.09 ± 0.01, 0.08 ± 0.01 and 0.09 ± 0.01 kg m^−2^ year^−1^, respectively). Sediment reworking rates were typically lower than that of bioerosion rates, except in the Nearshore Lagoon – Z3 (0.01 ± 0.002 kg m^−2^ year^−1^) and the *Porites* bommie – Z6 habitats.

When factoring for habitat scale, it was estimated that 44% of total sediment produced by parrotfish on the Vavvaru platform was produced in the Rubble habitat – Z5 (87,775 ± 11,151 kg year^−1^). Meanwhile, parrotfish in the two other habitats with high production rates, the NE reef – Z2 and SE patch reefs – Z1, only contributed 22% (44,814 ± 4908 kg year^−1^) and 6% (12,759 ± 1174 kg year^−1^) to the total supply of parrotfish‐derived sediment to the platform, respectively. Parrotfish in the hardground habitat also contributed a significant quantity of sediment (20% – 40,067 ± 7647 kg year^−1^) to the platform. Total sediment production and sediment reworking rates (and associated errors) by different sizes of Vavvaru parrotfish species can be found in Tables S2–S13.

### 3.2 Parrotfish contributions to sediment reworking

**FIGURE 3:**
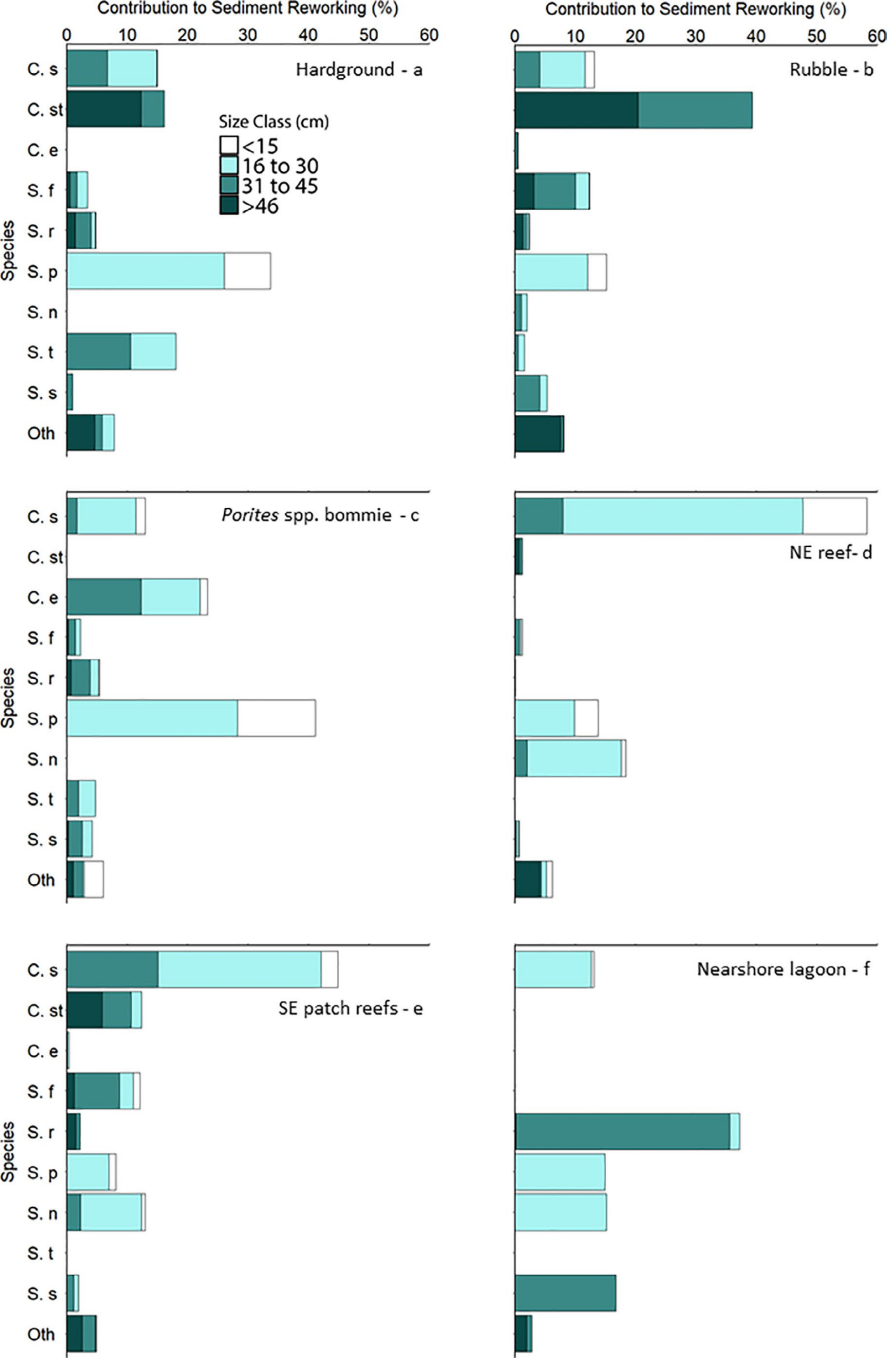
Percent contributions to total parrotfish sediment reworking by four size classes of fifteen species present in the six Vavvaru habitats supporting parrotfish: Species abbreviations: C. s – *Chlorurus sordidus*, C. st – *C. strongylocephalus*, C. e – *C. enneacanthus*, S. f – *Scarus frenatus*, S. r – *S. rubroviolaceus*, S. p – *S. psittacus*, S. n – *S. niger*, S. t – *S. tricolor*, S. s – *S. scaber*, Oth‐ Other species pooled

Scrapers were the dominant contributors to sediment reworking in the Hardground – Z4, *Porites* bommie – Z6, and Nearshore lagoon – Z3 habitats (contributing to 68%, 64%, and 87% in these habitats, respectively), while excavators were dominant in the Rubble – Z5, NE reef – Z2, and SE patch reef – Z1 habitats (58%, 64%, and 61%, respectively; Table 2). The dominant species and size classes that contributed to sediment reworking differed between habitats (Figure 3). *Scarus psittacus* was the dominant sediment reworker in the Hardground – Z4 (34%; 0.03 ± 0.005 kg m^−2^ year^−1^) and *Porites* bommie – Z6 (41%; 0.04 ± 0.006 kg m^−2^ year^−1^) habitats, *C. strongylocephalus* in the Rubble – Z5 habitat (39%; 0.03 ± 0.006 kg m^−2^ year^−1^), *C. sordidus* in the NE reef – Z2 (58%; 0.24 ± 0.06 kg m^−2^ year^−1^) and SE patch reef habitats – Z1 (45%; 0.13 ± 0.03 kg m^−2^ year^−1^), and *S. rubroviolaceus* in the Nearshore Lagoon – Z3 (37%; 0.005 ± 0.002 kg m^−2^ year^−1^). Note that species contributions to bioerosion (and therefore new sediment production from bioerosion) at Vavvaru are presented in Yarlett et al. (2020).

**TABLE 2:** Contributions to sediment reworking (% of total sediment reworked) by scrapers and excavators in the eight reef habitats at Vavvaru

Feeding mode	Z1	Z2	Z3	Z4	Z5	Z6
Excavators	61%	64%	13%	32%	58%	36%
Scrapers	39%	36%	87%	68%	42%	64%

### 3.3 Endogenous carbonate production

Endogenous carbonate samples examined under SEM revealed the presence of spheroid and rhombohedrons (sensu Salter et al., 2012) in starved scraper and excavator parrotfish (see Figure S2). However, despite thorough searching, these types of carbonates were not found at all under SEM in fecal samples of wild feeding parrotfish. The contribution of endogenous carbonates to new sediment production was, therefore, considered to be insignificant compared to that of eroded reef framework and was excluded from estimates of total sediment production.

### 3.4 Sediment grain size distribution and grain origins

**FIGURE 4:**
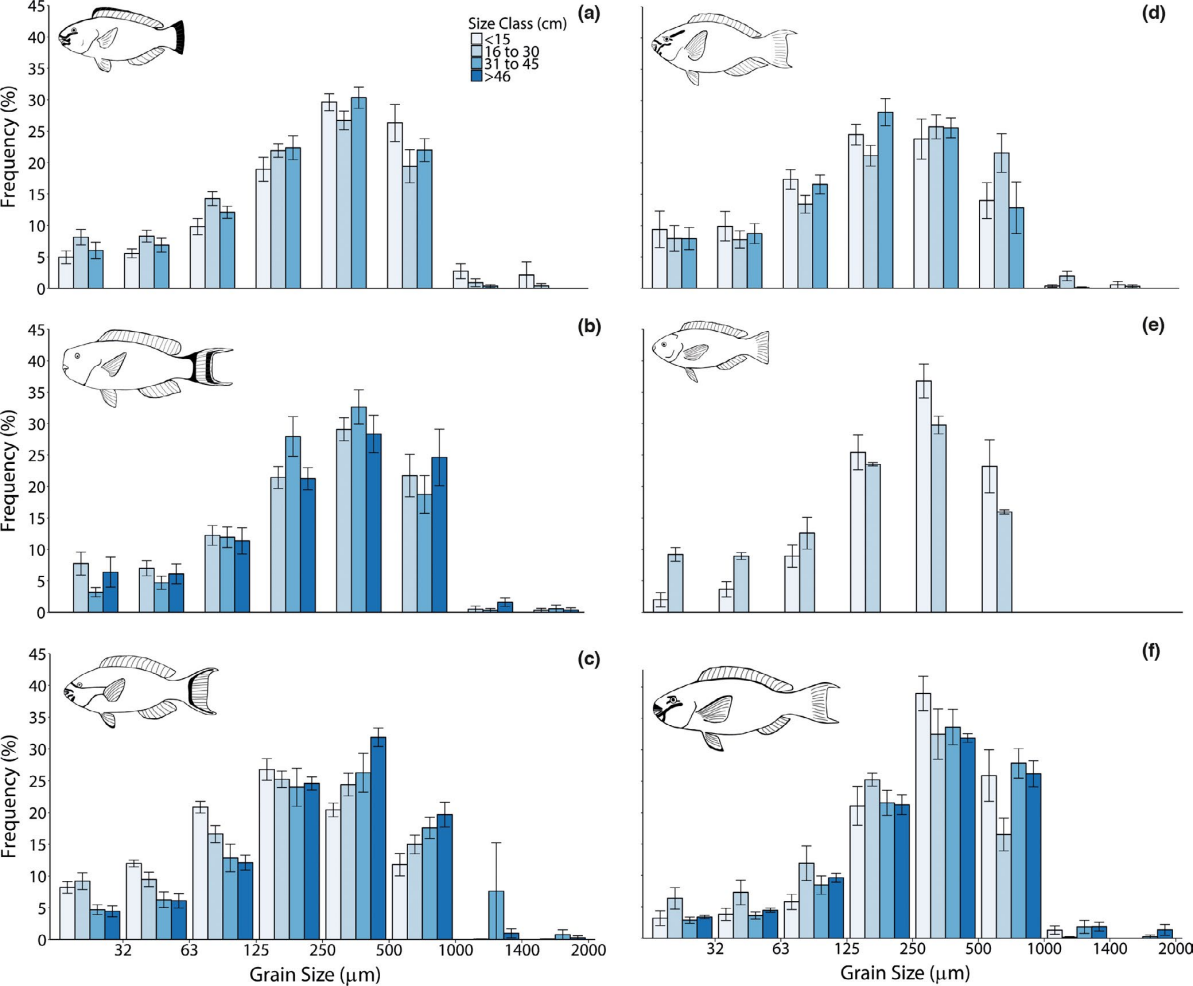
Grain size distributions of parrotfish derived sediments from excavators; (a) *Chlorurus sordidus*, and (b) *C. strongylocephalus*, and scrapers; (c) *Scarus frenatus*, (d) *S. niger*, (e) *S. psittacus*, (f) *S. rubroviolaceus*. For values and errors, see Tables S14–S19

**FIGURE 5:**
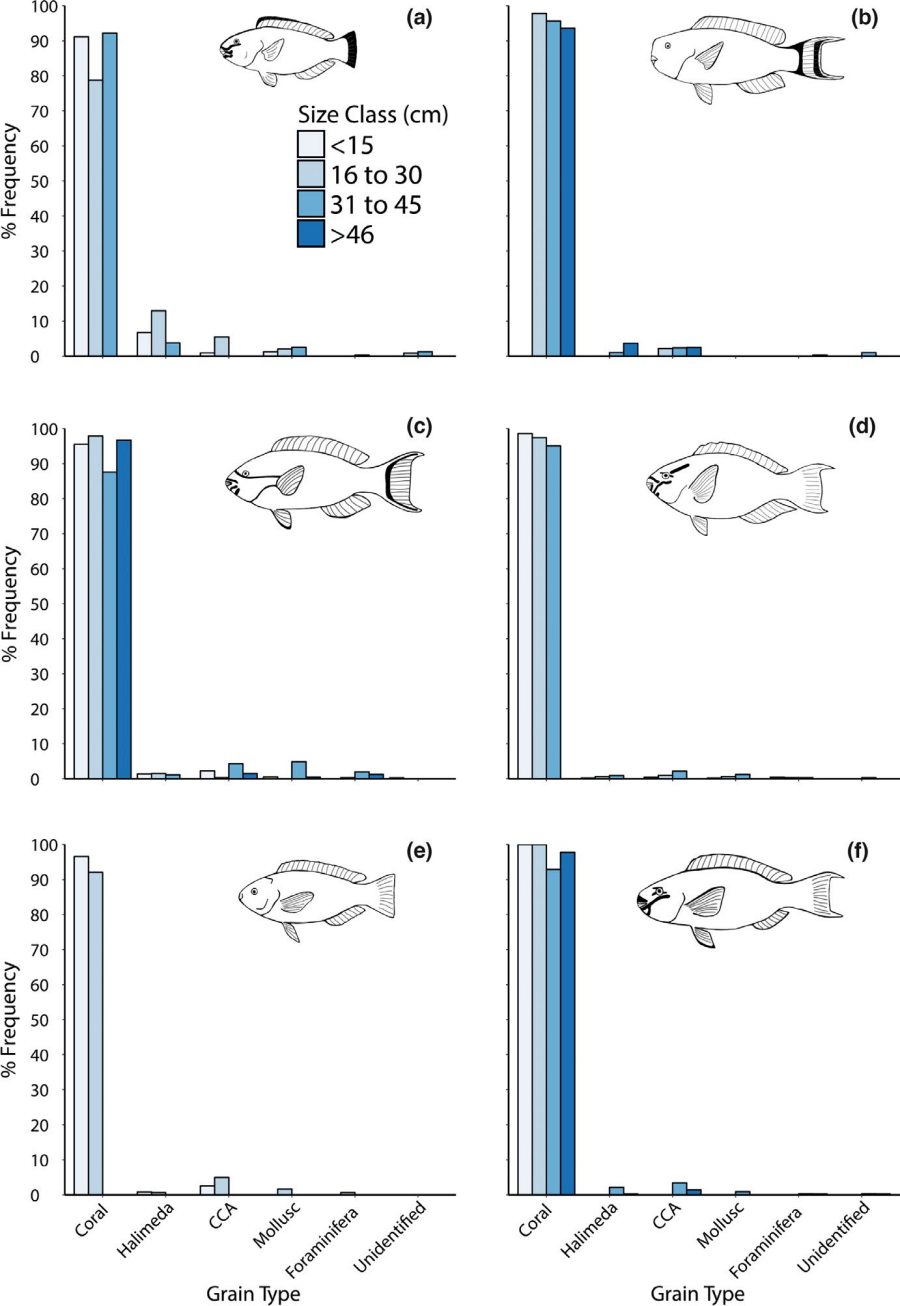
Origins of sediments produced by different size classes of six Maldivian parrotfish species; (a) *Chlorurus sordidus*, (b) *C. strongylocephalus*, (c) *S. frenatus*, (d) *S. niger*, (e) *S. psittacus*, (f) *S. rubroviolaceus*. Summary data are presented in Tables S20–S25

All species and sizes of parrotfish investigated produced a wide range of sediment size fractions, from silt to coarse sands (<32–2000 µm; Figure 4). The frequency of grains in all species gradually increased from fine size fractions (<63 µm) to peaks occurring between 125 and 1000 µm, but typically peaked in the 250–500 µm size fraction. There were few grains over 1000 µm but grains up to 2000 µm were present in small numbers in some species. No consistent relationship was found between fish size class and average sediment grain size in five of the six species studied, with average (median ‐ D_50_) grain sizes typically between 300 and 500 µm (Table 3). However, in the species *S. frenatus*, average (D_50_) grain size increased with fish body size, from 281.2 µm in <15 cm individuals to 515.5 µm in >45 cm individuals. Parrotfish fecal sediments originated primarily from coral skeletons (typically >80%), with a small percentage (typically <20%) of grains originating from *Halimeda* spp., Crustose Coralline Algae (CCA), foraminifera, mollusk shell fragments, and grains from unidentified origins (Figure 5). This aligned with parrotfish feeding preferences (derived from data collected in Yarlett et al., 2018) that show >95% of bites were taken on dead coral and coral rubble substrates, while few bites (typically <5%) were taken directly on *Halimeda* spp., CCA or live coral (Table 4).

**TABLE 3:** Descriptive statistics of sediment grain sizes produced by different parrotfish size classes

Species	Size class	D10 (µm)	D50 (µm)	D90 (µm)	Sorting (σ)
*C. s*	<15 cm	88.46	567.9	1310.4	2.614
	16–30 cm	78.28	340.4	1203.4	2.910
	31–45 cm	83.09	515.6	1207.9	2.609
	>46 cm	N/A	N/A	N/A	N/A
*C. st*	<15 cm	88.46	567.9	1310.4	2.614
	16–30 cm	80.27	509.8	1214.0	2.657
	31–45 cm	133.4	512.1	1188.1	2.455
	>46 cm	83.88	531.4	1254.1	2.637
*S. f*	<15 cm	75.18	281.2	1052.3	2.590
	16–30 cm	76.25	306.7	1121.4	2.624
	31–45 cm	87.21	515.1	1357.4	2.721
	>46 cm	88.30	515.5	1205.7	2.546
*S. n*	<15 cm	75.79	302.2	1124.4	2.634
	16–30 cm	79.04	353.1	1240.9	2.704
	31–45 cm	78.03	308.0	1080.7	2.570
	>46 cm	N/A	N/A	N/A	N/A
*S. p*	<15 cm	149.3	549.9	1211.1	2.179
	16–30 cm	76.69	333.1	1133.8	2.627
	31–45 cm	N/A	N/A	N/A	N/A
	>46 cm	N/A	N/A	N/A	N/A
*S. r*	<15 cm	150.7	578.1	1250.2	2.212
	16–30 cm	81.82	350.9	1146.0	2.562
	31–45 cm	145.4	575.7	1272.1	2.242
	>46 cm	135.5	565.9	1283.0	2.504

Note

*C*. *s – Chlorurus sordidus*, *C*. *st – Chlorurus strongylocephalus*, *S*. *f – Scarus frenatus*, *S*. *n – Scarus niger*, *S*. *p – Scarus psittacus*, *S*. *r – Scarus rubroviolaceus*.

**TABLE 4:** Percentage of parrotfish bites on dead coral or rubble, live coral, *Halimeda* spp., sand, and Crustose Coralline Algae (CCA) by different size classes of six parrotfish species

Species	Size class (cm)	Dead coral/rubble % bites	Live coral % bites	*Halimeda* spp. % bites	Sand % bites	CCA % bites
*C. sordidus*	<15	98.89	0.24	0.88	0.00	0.00
	16–30	98.31	0.44	1.26	0.00	0.00
	31–45	98.90	0.22	0.83	0.00	0.05
	>46	N/A	N/A	N/A	N/A	N/A
*C. strongylocephalus*	<15	95.61	0.00	4.39	0.00	0.00
	16–30	98.34	0.70	0.00	0.00	0.96
	31–45	99.75	0.11	0.00	0.07	0.07
	>46	99.56	0.44	0.00	0.00	0.00
*S. frenatus*	<15	94.93	0.15	1.88	0.00	3.04
	16–30	98.24	0.42	1.07	0.00	0.27
	31–45	97.69	1.07	0.72	0.03	0.49
	>46	100	0.00	0.00	0.00	0.00
*S. niger*	<15	98.35	0.33	0.87	0.00	0.46
	16–30	97.10	0.78	1.11	0.32	0.69
	31–45	98.87	0.33	0.66	0.10	0.03
	>46	N/A	N/A	N/A	N/A	N/A
*S. psittacus*	<15	97.67	0.25	1.12	0.56	0.40
	16–30	99.74	0.12	0.14	0.00	0.00
	31–45	N/A	N/A	N/A	N/A	N/A
	>46	N/A	N/A	N/A	N/A	N/A
*S. rubroviolaceus*	<15	98.55	1.45	0.00	0.00	0.00
	16–30	98.78	1.22	0.00	0.00	0.00
	31–45	99.30	0.13	0.00	0.57	0.00
	>46	99.89	0.11	0.00	0.00	0.00

Note

These data were collected during the bite rate measurements described in Yarlett et al. (2018) by recording the substrate that each bite was taken from but are reported here for comparison to sediment grain types produced by these species.

## 4 DISCUSSION

To protect the crucial role of parrotfish in reef carbonate and sediment budgets, it is important to understand how feeding by different parrotfish species assemblages, influenced by habitat type, translates to the rates of key functional roles. This is especially the case given the growing body of evidence for the role of parrotfish sediment production in shoreline sediment supply in some reef‐building regions (Morgan & Kench, 2016; Perry et al., 2015). Our findings show that local parrotfish species assemblages can have a significant influence on the total rate of parrotfish sediment production and determine whether this is new sediment (resulting from bioerosion of reef framework), or reworked existing sediment. In addition, we show that all species studied produce sediment size fractions suitable for shoreline maintenance.

The finding that parrotfish assemblages supported by the rubble – Z5 habitat produced the greatest quantity of new sediment (resulting from bioerosion of reef substrate, making up >90% of parrotfish‐derived sediment produced in the habitat) is an important finding because it demonstrates that naturally low coral cover habitats (see habitat summary data in Perry et al., 2017) can support species that perform important ecological functions. This is especially the case as some previous research has suggested parrotfish do not play an important role in rubble habitats in other reef building regions (Adam et al., 2015). It also reiterates the significant role that large excavating parrotfish play in supplying new sediment to reef habitats (Bellwood, 1996; Morgan & Kench, 2016; Ong & Holland, 2010), as bioerosion in this habitat was dominated by large (>30 cm) *C. strongylocephalus*. The atoll margin position of the Rubble – Z5 habitat may also add to its importance as a sediment source for the Vavvaru platform. Wave energy (required to entrain and transport coarse sediments) is likely to be consistently higher on the western atoll‐edge side of the platform, especially when strong monsoon winds (averaging 5.1 m s^−1^) blow from the west during April to November (Kench et al., 2006). These physical processes may have also influenced the sediment load in the epilithic algal matrix (EAM) observed in different habitats at Vavvaru. The eastern reef habitats retained nearly double the amount of sediment as the western habitats (~0.02 compared to ~0.01 g cm^3^, Table S26), which may have been caused by the physical transport (wind and wave energy) of sediments from the more exposed atoll edge (west – north west) toward the atoll lagoon side (east – south east) of the platform. This may also be driving a south easterly movement of Vavvaru Island itself (Perry et al., 2017).

These variations in sediment load may have influenced the parrotfish species found in each habitat, and as a result, influenced sediment reworking rates (Gordon et al., 2016). The highest sediment reworking rates were found in the eastern reef habitats (Z1 & 2) of Vavvaru, where reworking accounted for over 34% of parrotfish sediment production. This process formed a significant part of the high total sediment production rate in these habitats (>0.8 kg m^−2^ year^−1^), which was as high as the Rubble habitat – Z5. Sediment reworking differs from sediment production resulting from bioerosion in the sense that existing sediment is ingested, transported and re‐deposited, and so is not a source of new sediment to the reef. However, sediment reworking by parrotfish, as well as other fish groups such as some sediment ingesting surgeonfish, can complement abiotic factors as substrate “cleaning agents” (Goatley & Bellwood, 2010; Hubbard et al., 1990; Krone et al., 2011). Thus, in the eastern reef habitats (Z1 & 2), sediment reworking may be particularly important for clearing space to promote coral recruitment and sustain the high percentage coral cover observed in these habitats (Perry et al., 2017). The higher sediment loads in eastern reef habitat substrates did not appear to deter parrotfish feeding as reported for very high sediment loads in previous studies (Bellwood & Fulton, 2008; Bonaldo & Bellwood, 2011). However, whether the sediment load and grain size in these habitats influenced the species assemblage present requires further study (Gordon et al., 2016).

The proportion of sediment derived from bioerosion and from reworked existing sediment varied between species, functional group (scraper or excavator), and size classes of parrotfish, and total habitat production rates were influenced by reef habitat type. There are several factors likely playing a role in the patterns observed in the present study. The physical environment (e.g., topographic complexity, exposure to wave energy and currents) is likely to have an influence on species found in each habitat (Darling et al., 2017; Friedlander & Parrish, 1998; Graham & Nash, 2013; McClanahan & Author, 2001). For example, large excavators often prefer open environments near the reef slope, while smaller species often show preference for topographically complex habitats (Johnson et al., 2019). However, availability of preferred feeding substrate and substrate taphonomy is also likely to play an important role in parrotfish habitat preferences, bite rate, and resulting sediment production rate. Recent research has revealed evidence of trophic resource partitioning in several Indo‐Pacific parrotfish species, showing that different species selected different feeding substrata based on the successional stage of the substratum taphonomy and epilithic and endolithic biota (Nicholson & Clements, 2021). The preferred feeding substrate of parrotfish in the present study (dead coral and coral rubble substrates – representing over 95% of bites) can vary considerably in terms of hardness and extent of bioerosion (Scoffin, 1992), and is likely to drive differences in the substrates parrotfish feed on. For example, *Chlorurus microrhinos* (sister species of *C. strongylocephalus*) showed a preference for highly bioeroded, long dead coral (Nicholson & Clements, 2021). This was the dominant substrate type in the rocky rubble – Z5 habitat in the present study (Perry et al., 2017) and may explain the significant feeding activity and resultant sediment production by *C. strongylocephalus* in this habitat. The *Porites* spp. bommie habitat, on the other hand, lacks this type of substrate, being characterized by a limestone pavement substrate and sparse dead corals (Perry et al., 2017). This may explain why *C. enneacanthus* became the dominant bioeroder in this habitat – the only major reef habitat at Vavvaru where this role is not performed by either *C. strongylocephalus* or *C. sordidus* (Yarlett et al., 2020).

The process of sediment reworking may also contribute to the loss of sediment from the system by enhancing physical transport and reducing the grain size of sediments (Bellwood, 1996). This is partly because of active transport by parrotfish, but also because it causes fine sediments to become re‐suspended, where they may be more susceptible to hydrodynamic transport (Bellwood, 1996). The distance that these sediments travel depends on their grain size, shape, and density, as well as the distance from the seabed that they are egested and local current regimes (Bellwood, 1996; Kench, 1997, 1998; Kench & McLean, 1996). Previous work has estimated that fine sediments (<63 µm) suspended at 2 m above the seafloor could travel several hundred meters under gentle (~10 cm s^−1^) current regimes before settling (Bellwood, 1996). On a reef platform such as Vavvaru, which is only ~1000 m in diameter, it is likely that much of this material could be exported from the platform, particularly on the atoll‐edge (west) side of the platform, which experiences strong currents during changes in tidal state. Perimeter habitats may also be more susceptible to loss of sediment (and rubble) because of physical and hydrodynamic processes acting at the edge of the platform and reef slopes (Morgan & Kench, 2016b; Morgan et al., 2016).

Grain size distributions were comparable between all species studied but *S. psittacus* was the only species observed to not produce any grains over 1000 µm in diameter, likely reflected its weaker jaws, shallow bites, and limited bioerosion capability compared to the larger species studied (Bellwood & Choat, 1990; Nicholson & Clements, 2021). Only large excavators were likely to make meaningful contributions to very coarse grade sand production to the reef because of their high sediment production rates, especially given the small percentage of these size fractions (>1000 µm) produced. Interestingly, in most species we observed little difference in the grain sizes produced by parrotfish of different size classes. Our study is the first to examine this to the author's knowledge. *Scarus frenatus* was an exception as average grain size was observed to increase with body size. If this trend is apparent in more species (only 6 out of 15 parrotfish species were examined in detail at Vavvaru alone) then parrotfish size class distribution may influence the total quantity of different size fractions of sediment generated by whole parrotfish assemblages, which over larger scales, may influence the quantity of sediment that is retained or exported from the reef. Our parrotfish grain size distribution data follow a similar pattern to that observed by Morgan and Kench (2016a) for *C. strongylocephalus* in the Maldives using the same method. However, we observed a higher percentage (up to 20%, compared to <5%) of fines (all grain sizes <125 µm) and a less exaggerated peak in the size fraction between 250 and 500 µm (~30% compared to ~50%). The present study also observed a comparable proportion of grains originating from coral skeletons (>90%) to Morgan and Kench (2016a) in parrotfish‐derived sediments, but we observed a greater variety of other grain origins in small numbers (<5%), including foraminifera, mollusk shells, and *Halimeda* spp. in addition to crustose coralline algae. This reflected observed parrotfish feeding preferences (Table 4).

In the present study, extra care was taken to select samples where sediment egestion occurred close to the seabed to reduce dispersal of fines. However, it should be noted that some dispersal of fine sediments may have occurred during fecal sample collection. The proportion of fines is therefore thought to be a conservative estimate. However, we note that comparable work, albeit based on fish harvesting and gut content analysis, by Hoey and Bellwood (2008) on the Great Barrier Reef shows a similar grain size distribution for *C*. *microrhinos* (a sister species of *C. strongylocephalus* in the Pacific Ocean) as *C. strongylocephalus* in the present study. Data for *C. sordidus* (now *C*. *spilurus* in the Pacific) and *Scarus* spp. in the Hoey and Bellwood (2008) study returned a higher proportion (up to 30%) of fines (<63 µm), compared to ~10–20% in the present study. The fecal pellet methodology, whilst probably returning conservative estimates for fine‐grained sediment generation, can be inferred to return reasonable estimates of grain size distribution without the need to kill sample fish.

Another limitation of using the fecal pellet methodology for measuring grain size distributions of parrotfish sediments is the potential for contamination by benthic sediments. This was minimized in the present study by carefully choosing which samples to collect (e.g., relatively intact pellets egested close to “clean” substrates – live corals often worked well for this). The volume of sediment in intact pellets compared to that settled on the underlying substrate, combined with careful pipetting, meant that any contamination by benthic sediments was expected to be minimal. Analysis of benthic sediments at Vavvaru adds confidence to this as peaks in grain size typically occurred at smaller size fractions compared to parrotfish sediment (<250 µm compared to <500 µm, Figure S3).

The sediment grain sizes produced by parrotfish in the present study are comparable to those on some sand‐dominated Maldivian reef islands and beaches, particularly grains of coral origin on the beach toe (perimeter) area of islands (Morgan & Kench, 2016a; Perry et al., 2015). While the ultimate depositional fate of this sediment will depend on prevailing hydrodynamic (waves, currents) conditions, our study demonstrates that parrotfish do produce appropriate sediment types for shoreline sediment supply. Beach and island settings have been reported to have a more diverse grain composition than the parrotfish sediments observed in the present study (Perry et al., 2015), but parrotfish are likely to act as significant contributors of specifically coral sands to reef island systems in the central Indian Ocean.

We also found that unfed parrotfish did produce spheroid and rhombohedral carbonates endogenously, which originate from the precipitation of carbonates in parrotfish intestines as a by‐product of osmoregulation (Perry, Kench, et al., 2011; Walsh et al., 1991; Wilson et al., 1996, 2009; Wilson & Grosell, 2003). However, no traces of these carbonates were found when examining sediments produced by wild feeding parrotfish under SEM. Parrotfish intestines have been reported to be weakly acidic when feeding, with a pH of 6.4 recorded in parts of the intestine of *Scarus gibbus* (now *C. microrhinos*) (Smith & Paulson, 1974). However, extensive carbonate dissolution is unlikely because of the build‐up of CO_2_ this would cause. In addition, we observed no evidence of dissolution textures on the surface of carbonate grains in the parrotfish fecal samples examined in the present study. It may be that parrotfish do produce endogenous carbonates when feeding but these are dispersed rapidly upon entering the environment, so were not detected when collecting fecal samples. Whether or not scraper and excavator parrotfish produce endogenous carbonates when feeding remains unclear, but if they do not produce endogenous carbonates when feeding, it does pose an interesting biological question as to how they process excess calcium ingestion and prevent renal stone formation. For the purposes of the present study, it was clear that endogenous carbonate production was unlikely to be a relevant source of new sediment compared to that produced from eroded framework, so it was not included in estimates of total sediment production.

In addition to the themes already mentioned, we identified two further areas for future research. Firstly, studies to investigate the settling velocity of parrotfish‐derived sediments would be beneficial to make estimates of transport potential from suspension at different egestion distances from the seabed. This could be complimented with experiments to examine the current velocity required to entrain sediments of different types and size fractions to help predict the fate of sediments post‐egestion (Kench, 1997, 1998; Kench & McLean, 1996). This will be particularly relevant for predicting the proportions of sediment retention and export from the reef under scenarios of projected future sea level rise, especially as many Indian Ocean coral reefs are currently struggling to keep pace (Perry et al., 2018). Increased water depth above the reef platform is likely to increase both current speeds and wave energy, and therefore increase the chance of sediment hydrodynamic transport (Storlazzi et al., 2011). Secondly, future work on parrotfish functional roles would benefit from quantifying the amount of eroded material or loose sediment that is disturbed, but not ingested. This is different to the winnowing behavior observed in other sediment ingesting fish (e.g., Weller et al., 2016), which is not observed in parrotfish. On occasion, excavator parrotfish were anecdotally observed to break off tips of branching corals and drop the fragments onto the seabed. Parrotfish may, therefore, also produce coarser (gravel) grades of sediment through this process.

## 5 CONCLUSIONS

The present study furthers our understanding of how habitat type can influence total parrotfish sediment production rates (i.e., through bioerosion, sediment reworking, and endogenously produced sediment) resulting from the different species and size classes associated with those habitats. Understanding the habitats and species most important for parrotfish sediment production may help us to predict the implications arising from ongoing environmental disturbances on Indian Ocean reefs. For example, large *C. strongylocephalus* have been shown to undergo major declines following fishing pressure in some regions (Bellwood et al., 2012). In habitats like the Rubble – Z5 and Hardground – Z4 habitats in the present study, this could have profound consequences for new sediment production on coral reefs and shoreline maintenance on associated reef islands. Another threat to reef fish assemblages, including parrotfish, is a loss of habitat topographic complexity, often as a result of physical storm and wave damage or persistent erosion following coral mortality (Coker et al., 2012; Darling et al., 2017; Graham, 2014; Graham & Nash, 2013; Heenan et al., 2016; Richardson, Graham, Hoey, 2017; Richardson, Graham, Pratchett et al., 2017). In the eastern reef habitats in the present study, some of the most important sediment reworkers, such as *C. sordidus* and *S. niger*, appeared to associate with more topographically complex habitats (Yarlett et al., 2020). A loss of structure in comparable habitats may cause declines in these species and their sediment reworking (and therefore substrate cleaning) function. This may lead to a reduction in coral recruitment success and opportunities for habitat recovery (Goatley & Bellwood, 2010; Krone et al., 2011).

## CONFLICT OF INTEREST

The authors declare that they have no conflict of interest.

## AUTHOR CONTRIBUTIONS

**Robert T. Yarlett:** Conceptualization (equal); Formal analysis (lead); Funding acquisition (equal); Investigation (lead); Methodology (lead); Writing‐original draft (lead); Writing‐review & editing (lead). **Chris T. Perry:** Conceptualization (equal); Funding acquisition (equal); Investigation (equal); Methodology (equal); Supervision (lead); Writing‐review & editing (equal). **Rod W. Wilson:** Conceptualization (equal); Investigation (equal); Methodology (equal); Supervision (equal); Writing‐review & editing (equal).

## Supporting information

Supplementary Material

## Data Availability

Raw data files can be found at https://doi.org/10.24378/exe.3563. Summary data can also be found in the Electronic Supplementary Material.
