# Supplementary material for: Quantifying production rates and size fractions of parrotfish‐derived sediment: A key functional role on Maldivian coral reefs
Source: Ecol Evol. 2021 Nov 3;11(22):16250–65. doi: 10.1002/ece3.8306 (PMC8601892; doi:10.1002/ece3.8306)
Supplement: Supplementary file 1 — Supplementary Material [file ECE3-11-16250-s001.pdf]

Electronic Supplementary Information

Statistics and Error Propagation

Some of the calculations in the present study involved summing or multiplying variables, such as for calculating species and habitat scale sediment reworking and total sediment production rates, each with their own associated standard errors. In these calculations, the following rules for error propagation were used.

For multiplication:

$$SEv = \sqrt{\left(\frac{SEx}{\bar{x}}\right)^2 + \left(\frac{SEy}{\bar{y}}\right)^2} \times \bar{v}$$

Where $\bar{v}$ = calculated value, x = variable 1 and y = variable 2.

For addition and subtraction:

$$SEv = \sqrt{(SEx1)^2 + (SEx2)^2 + \ldots + (SExn)^2}$$

Images of Parrotfish Sediments

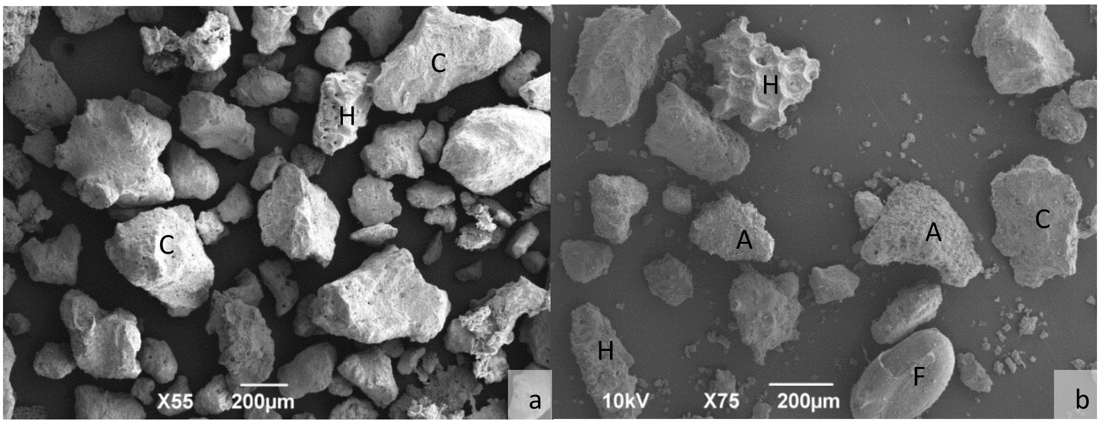

Fig. S1: (a, b) SEM images of parrotfish faecal sediments, with examples of common grain types; C - Coral, H - *Halimeda* spp., A - Coralline Algae and F - Foraminifera.

Endogenous Carbonate Images

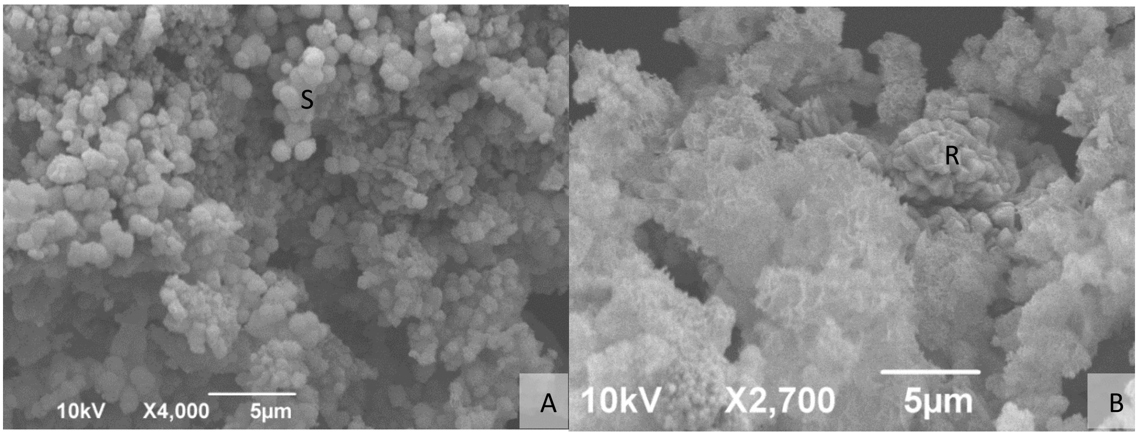

Fig. S2: SEM images of endogenous carbonate grain morphologies; S – Speheroids (in A) and R – Rhomboids (in B) produced in non-feeding parrotfish.

Supplementary Table 1: Data assumptions for where species data are absent. Data source is Yarlett *et al.* (2018) - MEPS

Species	Data assumption
Chlorurus enneacanthus	*Chlorurus sordidus*
Scarus tricolor	*Scarus niger*
Scarus scaber	*Scarus frenatus*
Scarus prasiognathos	*Scarus frenatus*
Scarus viridifucatus	*Scarus frenatus*
Scarus russelii	*Scarus frenatus*
Hipposcarus harid	*Scarus frenatus*
Cetoscarus ocellatus	*Chlorurus strongylocephalus*
Juveniles	Lowest measured bioerosion rate at < 15 cm

Data Tables for Total Sediment Production and Sediment Reworking Rates

The following data tables provide the annual rates and standard errors of total sediment production and sediment reworking rates for different size classes of all parrotfish species observed at Vavvaru. **All rates are in g m^{-2} yr^{-1}.**

Supplementary Table 2: Sediment reworking rates for Vavvaru parrotfish in the Hardground – Z4 habitat.

**Hardground**	**Fish Size**			
**(g m^{-2} yr^{-1})**	**1 to 15 cm**	**16 to 30 cm**	**31 to 45 cm**	**46 to 60 cm**
Chlorurus sordidus	0.05 ± 0.01	7.04 ± 1.36	5.81 ± 0.79	0.00 ± 0.00
Chlorurus strongylocephalus	0.00 ± 0.00	0.00 ± 0.00	3.30 ± 0.72	10.61 ± 3.45
Chlorurus enneacanthus	0.00 ± 0.00	0.03 ± 0.01	0.00 ± 0.00	0.00 ± 0.00
Scarus frenatus	0.00 ± 0.00	1.48 ± 0.45	1.02 ± 0.22	0.44 ± 0.11
Scarus rubroviolaceus	0.09 ± 0.04	0.60 ± 0.16	2.25 ±0.66	1.19 ± 0.35
Scarus psittacus	6.63 ± 1.20	22.41 ± 4.36	0.00 ± 0.00	0.00 ± 0.00
Scarus niger	0.00 ± 0.00	0.05 ± 0.02	0.00 ± 0.00	0.00 ± 0.00
Scarus tricolor	0.00 ± 0.00	6.46 ± 1.12	9.06 ± 1.98	0.00 ± 0.00
Scarus scaber	0.00 ± 0.00	0.04 ± 0.01	0.84 ± 0.30	0.00 ± 0.00
Scarus prasiognathos	0.00 ± 0.00	0.00 ±0.00	0.00 ± 0.00	2.81 ± 0.58
Scarus viridifucatus	0.00 ± 0.00	0.00 ± 0.00	0.00 ±0.00	0.00 ± 0.00
Scarus russelii	0.00 ± 0.00	1.74 ±0.5	0.78 ± 0.14	0.00 ± 0.00
Hipposcarus harid	0.00 ± 0.00	0.00 ± 0.00	0.24 ± 0.07	0.56 ± 0.11
Cetoscarus ocellatus	0.00 ± 0.00	0.00 ±0.00	0.00 ± 0.00	0.63 ±0.50
Calotomus carolinus	0.00 ± 0.00	0.00 ± 0.00	0.00 ±0.00	0.00 ± 0.00
Juveniles	0.00 ± 0.00	0.00 ± 0.00	0.00 ±0.00	0.00 ± 0.00

Supplementary Table 3: Sediment reworking rates for Vavvaru parrotfish in the Rubble – Z5 habitat.

**Rubble**	**Fish Size**			
**(g m^{-2} yr^{-1})**	**1 to 15 cm**	**16 to 30 cm**	**31 to 45 cm**	**46 to 60 cm**
Chlorurus sordidus	1.19 ± 0.45	5.64 ± 1.86	3.10 ± 2.19	0.00 ± 0.00
Chlorurus strongylocephalus	0.00 ± 0.00	0.00 ± 0.00	14.24 ± 3.76	15.41 ± 4.77
Chlorurus enneacanthus	0.00 ± 0.00	0.09 ± 0.04	0.34 ± 0.27	0.00 ± 0.00
Scarus frenatus	0.04 ± 0.02	1.69 ±0.37	5.19 ± 1.47	2.40 ± 0.77
Scarus rubroviolaceus	0.08 ± 0.11	0.25 ± 0.13	0.47 ± 0.26	0.98 ± 0.36
Scarus psittacus	2.32 ± 1.13	9.11 ± 4.08	0.00 ± 0.00	0.00 ± 0.00
Scarus niger	0.00 ± 0.00	0.73 ± 0.56	0.81 ± 0.49	0.00 ± 0.00
Scarus tricolor	0.00 ± 0.00	0.80 ± 0.40	0.40 ± 0.24	0.00 ± 0.00
Scarus scaber	0.00 ± 0.00	0.95 ± 0.45	3.08 ± 0.88	0.00 ± 0.00
Scarus prasiognathos	0.00 ± 0.00	0.03 ± 0.01	0.14 ± 0.11	1.83 ± 0.59
Scarus viridifucatus	0.00 ± 0.00	0.00 ± 0.00	0.00 ± 0.00	0.00 ± 0.00
Scarus russelii	0.00 ± 0.00	0.00 ± 0.00	0.00 ± 0.00	0.00 ± 0.00
Hipposcarus harid	0.00 ± 0.00	0.00 ± 0.00	0.20 ± 0.08	0.30 ± 0.12
Cetoscarus ocellatus	0.00 ± 0.00	0.00 ± 0.00	0.00 ± 0.00	3.60 ± 1.28
Calotomus carolinus	0.00 ± 0.00	0.00 ± 0.00	0.00 ± 0.00	0.00 ± 0.00
Juveniles	0.03 ± 0.03	0.00 ± 0.00	0.00 ± 0.00	0.00 ± 0.00

Supplementary Table 4: Sediment reworking rates for Vavvaru parrotfish in the *Porites* spp. bommie – Z6 habitat.

**Porites bommie**	**Fish Size**			
**(g m^{-2} yr^{-1})**	**1 to 15 cm**	**16 to 30 cm**	**31 to 45 cm**	**46 to 60 cm**
Chlorurus sordidus	1.48 ± 0.40	9.09 ± 2.19	1.59 ± 0.96	0.00 ± 0.00
Chlorurus strongylocephalus	0.00 ± 0.00	0.00 ± 0.00	0.00 ± 0.00	0.00 ± 0.00
Chlorurus enneacanthus	1.19 ± 0.47	9.14 ± 1.71	11.50 ± 3.26	0.00 ± 0.00
Scarus frenatus	0.00 ± 0.00	0.85 ± 0.25	1.06 ± 0.30	0.23 ± 0.11
Scarus rubroviolaceus	0.12 ± 0.06	1.33 ± 0.45	2.96 ± 0.82	0.65 ± 0.28
Scarus psittacus	12.10 ± 3.12	26.45 ± 5.51	0.00 ± 0.00	0.00 ± 0.00
Scarus niger	0.00 ± 0.00	0.00 ± 0.00	0.00 ± 0.00	0.00 ± 0.00
Scarus tricolor	0.00 ± 0.00	2.64 ± 0.63	1.79 ± 0.60	0.00 ± 0.00
Scarus scaber	0.01 ± 0.00	1.56 ± 0.34	2.09 ± 0.47	0.27 ± 0.21
Scarus prasiognathos	0.00 ± 0.00	0.19 ± 0.11	1.25 ± 0.38	0.31 ± 0.08
Scarus viridifucatus	0.00 ± 0.00	0.00 ± 0.00	0.08 ± 0.06	0.00 ± 0.00
Scarus russelii	0.00 ± 0.00	0.00 ± 0.00	0.00 ± 0.00	0.00 ± 0.00
Hipposcarus harid	0.00 ± 0.00	0.03 ± 0.03	0.17 ± 0.13	0.66 ± 0.24
Cetoscarus ocellatus	0.00 ± 0.00	0.00 ± 0.00	0.00 ± 0.00	0.00 ± 0.00
Calotomus carolinus	0.00 ± 0.00	0.00 ± 0.00	0.00 ± 0.00	0.00 ± 0.00
Juveniles	2.95 ± 1.43	0.00 ± 0.00	0.00 ± 0.00	0.00 ± 0.00

Supplementary Table 5: Sediment reworking rates for Vavvaru parrotfish in the NE reef – Z2 habitat.

NE reef	Fish Size			
**(g m^{-2} yr^{-1})**	**1 to 15 cm**	**16 to 30 cm**	**31 to 45 cm**	**46 to 60 cm**
Chlorurus sordidus	43.11 ± 13.72	160.92 ± 54.24	32.24 ± 13.57	0.00 ± 0.00
Chlorurus strongylocephalus	0.00 ± 0.00	0.22 ± 0.33	2.08 ± 1.37	2.86 ± 1.97
Chlorurus enneacanthus	0.00 ± 0.00	0.00 ± 0.00	0.00 ± 0.00	0.00 ± 0.00
Scarus frenatus	1.03 ± 0.63	1.07 ±0.67	2.85 ± 1.68	0.13 ± 0.10
Scarus rubroviolaceus	0.00 ± 0.00	0.02 ± 0.04	0.39 ± 0.26	0.08 ± 0.07
Scarus psittacus	15.98 ± 7.89	40.05 ± 22.29	0.00 ± 0.00	0.00 ± 0.00
Scarus niger	2.98 ± 1.14	63.23 ± 26.30	8.24 ± 3.59	0.00 ± 0.00
Scarus tricolor	0.00 ± 0.00	0.15 ± 0.23	0.00 ± 0.00	0.00 ± 0.00
Scarus scaber	0.08 ± 0.07	1.53 ± 1.08	1.14 ± 1.31	0.00 ± 0.00
Scarus prasiognathos	0.01 ± 0.01	1.61 ± 0.76	0.00 ± 0.00	0.00 ± 0.00
Scarus viridifucatus	0.00 ± 0.00	1.91 ± 0.86	0.11 ± 0.08	0.00 ± 0.00
Scarus russelii	0.00 ± 0.00	0.00 ±0.00	0.00 ± 0.00	0.00 ± 0.00
Hipposcarus harid	0.00 ± 0.00	0.00 ± 0.00	0.00 ± 0.00	0.23 ± 0.12
Cetoscarus ocellatus	0.00 ± 0.00	0.00 ± 0.00	0.86 ± 0.48	16.55 ± 9.40
Calotomus carolinus	0.00 ± 0.00	0.00 ± 0.00	0.00 ± 0.00	0.00 ± 0.00
Juveniles	3.91 ± 4.00	0.00 ± 0.00	0.00 ± 0.00	0.00 ± 0.00

Supplementary Table 6: Sediment reworking rates for Vavvaru parrotfish in the SE patch reef – Z1 habitat.

SE patch reefs	Fish Size			
**(g m^{-2} yr^{-1})**	**1 to 15 cm**	**16 to 30 cm**	**31 to 45 cm**	**46 to 60 cm**
Chlorurus sordidus	8.37 ± 2.65	80.83 ± 21.43	44.93 ± 14.27	0.00 ± 0.00
Chlorurus strongylocephalus	0.00 ± 0.00	5.23 ± 4.22	14.27 ± 4.86	17.59 ± 6.53
Chlorurus enneacanthus	0.00 ± 0.00	0.71 ± 0.26	0.36 ± 0.33	0.00 ± 0.00
Scarus frenatus	3.32 ± 1.07	6.74 ± 1.68	22.59 ± 4.63	3.57 ± 1.48
Scarus rubroviolaceus	0.00 ± 0.00	0.12 ± 0.06	2.06 ± 0.77	4.47 ± 1.76
Scarus psittacus	3.30 ± 1.79	20.97 ± 11.30	0.00 ± 0.00	0.00 ± 0.00
Scarus niger	1.62 ± 0.46	30.32 ± 13.11	6.80 ± 3.87	0.00 ± 0.00
Scarus tricolor	0.00 ± 0.00	0.00 ± 0.00	0.00 ± 0.00	0.00 ± 0.00
Scarus scaber	0.00 ± 0.00	2.48 ± 0.79	3.32 ± 1.11	0.00 ± 0.00
Scarus prasiognathos	0.00 ± 0.00	0.00 ± 0.00	0.00 ± 0.00	0.00 ± 0.00
Scarus viridifucatus	0.00 ± 0.00	0.50 ± 0.37	0.17 ± 0.15	0.00 ± 0.00
Scarus russelii	0.00 ± 0.00	0.00 ± 0.00	0.00 ± 0.00	0.00 ± 0.00
Hipposcarus harid	0.00 ± 0.00	0.10 ± 0.03	1.77 ± 1.16	0.64 ± 0.31
Cetoscarus ocellatus	0.00 ± 0.00	0.00 ± 0.00	4.26 ± 1.23	6.97 ± 2.93
Calotomus carolinus	0.00 ± 0.00	0.00 ± 0.00	0.00 ± 0.00	0.00 ± 0.00
Juveniles	0.34 ± 0.19	0.00 ± 0.00	0.00 ± 0.00	0.00 ± 0.00

Supplementary Table 7: Sediment reworking rates for Vavvaru parrotfish in the Nearshore – Z3 habitat.

Nearshore lagoon	Fish Size			
**(g m^{-2} yr^{-1})**	**1 to 15 cm**	**16 to 30 cm**	**31 to 45 cm**	**46 to 60 cm**
Chlorurus sordidus	0.06 ± 0.06	1.87 ± 0.29	0.00 ± 0.00	0.00 ± 0.00
Chlorurus strongylocephalus	0.00 ± 0.00	0.00 ± 0.00	0.00 ± 0.00	0.00 ± 0.00
Chlorurus enneacanthus	0.00 ± 0.00	0.00 ± 0.00	0.00 ± 0.00	0.00 ± 0.00
Scarus frenatus	0.00 ± 0.00	0.00 ± 0.00	0.00 ± 0.00	0.00 ± 0.00
Scarus rubroviolaceus	0.00 ± 0.00	0.25 ± 0.18	5.21 ± 1.80	0.03 ± 0.02
Scarus psittacus	0.00 ± 0.00	2.20 ± 0.69	0.00 ± 0.00	0.00 ± 0.00
Scarus niger	0.00 ± 0.00	2.24 ± 0.51	0.00 ±0.00	0.00 ± 0.00
Scarus tricolor	0.00 ± 0.00	0.00 ± 0.00	0.00 ± 0.00	0.00 ± 0.00
Scarus scaber	0.00 ± 0.00	0.00 ± 0.00	2.47 ±0.50	0.00 ± 0.00
Scarus prasiognathos	0.00 ± 0.00	0.00 ± 0.00	0.00 ± 0.00	0.00 ± 0.00
Scarus viridifucatus	0.00 ± 0.00	0.00 ± 0.00	0.00 ± 0.00	0.00 ± 0.00
Scarus russelii	0.00 ± 0.00	0.00 ± 0.00	0.00 ± 0.00	0.00 ± 0.00
Hipposcarus harid	0.00 ± 0.00	0.00 ± 0.00	0.14 ± 0.05	0.28 ± 0.10
Cetoscarus ocellatus	0.00 ± 0.00	0.00 ± 0.00	0.00 ± 0.00	0.00 ± 0.00
Calotomus carolinus	0.00 ± 0.00	0.00 ± 0.00	0.00 ± 0.00	0.00 ± 0.00
Juveniles	0.00 ± 0.00	0.00 ± 0.00	0.00 ± 0.00	0.00 ± 0.00

Supplementary Table 8: Total sediment production rates for Vavvaru parrotfish in the Hardground – Z4 habitat.

**Hardground**	**Fish Size**			
**(g m^{-2} yr^{-1})**	**1 to 15 cm**	**16 to 30 cm**	**31 to 45 cm**	**46 to 60 cm**
Chlorurus sordidus	0.08 ± 0.02	16.81 ± 3.61	25.57 ± 2.63	0.00 ± 0.00
Chlorurus strongylocephalus	0.00 ± 0.00	0.00 ± 0.00	103.76 ± 25.57	330.54 ± 106.83
Chlorurus enneacanthus	0.00 ± 0.00	0.08 ± 0.02	0.00 ± 0.00	0.00 ± 0.00
Scarus frenatus	0.00 ± 0.00	2.03 ± 0.49	1.59 ± 0.26	0.69 ± 0.13
Scarus rubroviolaceus	0.11 ± 0.04	0.74 ± 0.17	5.33 ± 1.14	3.16 ± 0.65
Scarus psittacus	8.24 ± 1.26	32.35 ± 4.80	0.00 ± 0.00	0.00 ± 0.00
Scarus niger	0.00 ± 0.00	0.07 ± 0.02	0.00 ± 0.00	0.00 ± 0.00
Scarus tricolor	0.00 ± 0.00	9.33 ± 1.22	11.98 ± 2.11	0.00 ± 0.00
Scarus scaber	0.00 ± 0.00	0.06 ± 0.01	1.31 ± 0.35	0.00 ± 0.00
Scarus prasiognathos	0.00 ± 0.00	0.00 ± 0.00	0.00 ± 0.00	4.40 ± 0.67
Scarus viridifucatus	0.00 ± 0.00	0.00 ± 0.00	0.00 ± 0.00	0.00 ± 0.00
Scarus russelii	0.00 ± 0.00	2.39 ± 0.63	1.21 ± 0.15	0.00 ± 0.00
Hipposcarus harid	0.00 ± 0.00	0.00 ± 0.00	0.37 ± 0.08	0.88 ± 0.13
Cetoscarus ocellatus	0.00 ± 0.00	0.00 ± 0.00	0.00 ± 0.00	19.58 ± 15.65
Calotomus carolinus	0.00 ± 0.00	0.00 ± 0.00	0.00 ± 0.00	0.00 ± 0.00
Juveniles	0.00 ± 0.00	0.00 ± 0.00	0.00 ± 0.00	0.00 ± 0.00

Supplementary Table 9: Total sediment production rates for Vavvaru parrotfish in the Rubble – Z5 habitat.

**Rubble**	**Fish Size**			
**(g m^{-2} yr^{-1})**	**1 to 15 cm**	**16 to 30 cm**	**31 to 45 cm**	**46 to 60 cm**
Chlorurus sordidus	1.77 ± 0.50	11.90 ± 3.20	11.52 ± 6.03	0.00 ± 0.00
Chlorurus strongylocephalus	0.00 ± 0.00	0.00 ± 0.00	361.15 ± 75.28	387.40 ± 86.31
Chlorurus enneacanthus	0.00 ± 0.00	0.19 ± 0.06	1.27 ± 0.75	0.00 ± 0.00
Scarus frenatus	0.04 ± 0.02	2.20 ± 0.38	7.53 ± 1.55	3.48 ± 0.82
Scarus rubroviolaceus	0.10 ± 0.11	0.30 ± 0.13	0.99 ± 0.36	2.27 ± 0.54
Scarus psittacus	2.77 ±1.15	12.34 ± 4.24	0.00 ± 0.00	0.00 ± 0.00
Scarus niger	0.00 ± 0.00	0.99 ± 0.58	1.02 ± 0.50	0.00 ± 0.00
Scarus tricolor	0.00 ± 0.00	1.08 ± 0.42	0.50 ± 0.24	0.00 ± 0.00
Scarus scaber	0.00 ± 0.00	1.23 ± 0.47	4.47 ± 0.93	0.00 ± 0.00
Scarus prasiognathos	0.00 ± 0.00	0.04 ± 0.01	0.21 ± 0.12	2.65 ± 0.63
Scarus viridifucatus	0.00 ± 0.00	0.00 ± 0.00	0.00 ± 0.00	0.00 ± 0.00
Scarus russelii	0.00 ± 0.00	0.00 ± 0.00	0.00 ± 0.00	0.00 ± 0.00
Hipposcarus harid	0.00 ± 0.00	0.00 ± 0.00	0.29 ± 0.09	0.44 ± 0.13
Cetoscarus ocellatus	0.00 ± 0.00	0.00 ± 0.00	0.00 ± 0.00	90.55 ± 14.04
Calotomus carolinus	0.00 ± 0.00	0.00 ± 0.00	0.00 ± 0.00	0.00 ± 0.00
Juveniles	0.03 ± 0.03	0.00 ± 0.00	0.00 ± 0.00	0.00 ± 0.00

Supplementary Table 10: Total sediment production rates for Vavvaru parrotfish in the *Porites* bommie – Z6 habitat.

***Porites* bommie**	**Fish Size**			
**(g m^{-2} yr^{-1})**	**1 to 15 cm**	**16 to 30 cm**	**31 to 45 cm**	**46 to 60 cm**
Chlorurus sordidus	2.20 ± 0.45	19.18 ± 3.77	5.91 ± 2.63	0.00 ± 0.00
Chlorurus strongylocephalus	0.00 ± 0.00	0.00 ± 0.00	0.00 ± 0.00	0.00 ± 0.00
Chlorurus enneacanthus	1.77 ± 0.53	19.28 ± 2.93	42.81 ± 9.00	0.00 ± 0.00
Scarus frenatus	0.00 ± 0.00	1.10 ± 0.26	1.54 ± 0.31	0.34 ± 0.12
Scarus rubroviolaceus	0.14 ± 0.06	1.58 ± 0.46	6.21 ± 1.14	1.52 ± 0.42
Scarus psittacus	14.45 ± 3.17	35.83 ± 5.75	0.00 ± 0.00	0.00 ± 0.00
Scarus niger	0.00 ± 0.00	0.00 ± 0.00	0.00 ± 0.00	0.00 ± 0.00
Scarus tricolor	0.00 ± 0.00	3.57 ± 0.66	2.25 ± 0.61	0.00 ± 0.00
Scarus scaber	0.01 ± 0.00	2.02 ± 0.36	3.04 ± 0.49	0.39 ± 0.22
Scarus prasiognathos	0.00 ± 0.00	0.24 ± 0.11	1.81 ± 0.40	0.45 ± 0.08
Scarus viridifucatus	0.00 ± 0.00	0.00 ± 0.00	0.12 ± 0.07	0.00 ± 0.00
Scarus russelii	0.00 ± 0.00	0.00 ± 0.00	0.00 ± 0.00	0.00 ±0.00
Hipposcarus harid	0.00 ± 0.00	0.04 ± 0.03	0.24 ± 0.14	0.96 ± 0.26
Cetoscarus ocellatus	0.00 ± 0.00	0.00 ± 0.00	0.00 ± 0.00	0.00 ± 0.00
Calotomus carolinus	0.00 ± 0.00	0.00 ± 0.00	0.00 ± 0.00	0.00 ± 0.00
Juveniles	3.38 ± 1.45	0.00 ± 0.00	0.00 ± 0.00	0.00 ± 0.00

Supplementary Table 11: Total sediment production rates for Vavvaru parrotfish in the NE reef – Z2 habitat.

NE Reef	Fish Size			
**(g m^{-2} yr^{-1})**	**1 to 15 cm**	**16 to 30 cm**	**31 to 45 cm**	**46 to 60 cm**
Chlorurus sordidus	54.17 ± 13.84	254.93 ± 58.08	78.42 ± 16.78	0.00 ± 0.00
Chlorurus strongylocephalus	0.00 ± 0.00	1.95 ± 1.48	28.72 ± 8.25	39.24 ± 11.87
Chlorurus enneacanthus	0.00 ± 0.00	0.00 ± 0.00	0.00 ± 0.00	0.00 ± 0.00
Scarus frenatus	1.08 ± 0.63	1.24 ± 0.67	3.52 ± 1.69	0.16 ± 0.10
Scarus rubroviolaceus	0.00 ± 0.00	0.02 ± 0.04	0.61 ± 0.27	0.14 ± 0.08
Scarus psittacus	17.62 ± 7.90	47.53 ± 22.36	0.00 ± 0.00	0.00 ± 0.00
Scarus niger	3.29 ± 1.14	75.04 ± 26.41	9.36 ± 3.60	0.00 ± 0.00
Scarus tricolor	0.00 ± 0.00	0.18 ± 0.23	0.00 ± 0.00	0.00 ± 0.00
Scarus scaber	0.09 ± 0.07	1.77 ± 1.08	1.41 ± 1.32	0.00 ± 0.00
Scarus prasiognathos	0.01 ± 0.01	1.86 ± 0.76	0.00 ± 0.00	0.00 ± 0.00
Scarus viridifucatus	0.00 ± 0.00	2.21 ± 0.86	0.14 ± 0.08	0.00 ± 0.00
Scarus russelii	0.00 ± 0.00	0.00 ± 0.00	0.00 ± 0.00	0.00 ± 0.00
Hipposcarus harid	0.00 ± 0.00	0.00 ± 0.00	0.00 ± 0.00	0.28 ± 0.12
Cetoscarus ocellatus	0.00 ± 0.00	0.00 ± 0.00	11.93 ± 2.33	226.81 ± 60.61
Calotomus carolinus	0.00 ± 0.00	0.00 ± 0.00	0.00 ± 0.00	0.00 ± 0.00
Juveniles	4.20 ± 4.01	0.00 ± 0.00	0.00 ± 0.00	0.00 ± 0.00

Supplementary Table 12: Total sediment production rates for Vavvaru parrotfish in the SE reef – Z1 habitat.

SE Patch Reefs	Fish Size			
**(g m^{-2} yr^{-1})**	**1 to 15 cm**	**16 to 30 cm**	**31 to 45 cm**	**46 to 60 cm**
Chlorurus sordidus	10.07 ± 2.67	118.21 ± 23.83	95.89 ± 19.44	0.00 ± 0.00
Chlorurus strongylocephalus	0.00 ± 0.00	38.13 ± 23.21	159.06 ± 39.06	194.45 ± 50.19
Chlorurus enneacanthus	0.00 ± 0.00	1.04 ± 0.29	0.77 ± 0.45	0.00 ± 0.00
Scarus frenatus	3.46 ± 1.07	7.58 ± 1.68	26.84 ± 4.67	4.24 ± 1.49
Scarus rubroviolaceus	0.00 ± 0.00	0.13 ± 0.06	3.00 ± 0.82	6.93 ± 1.90
Scarus psittacus	3.57 ± 1.80	24.07 ± 11.35	0.00 ± 0.00	0.00 ± 0.00
Scarus niger	1.75 ± 0.46	34.80 ± 13.19	7.53 ± 3.88	0.00 ± 0.00
Scarus tricolor	0.00 ± 0.00	0.00 ± 0.00	0.00 ± 0.00	0.00 ± 0.00
Scarus scaber	0.00 ± 0.00	2.78 ± 0.79	3.94 ± 1.12	0.00 ± 0.00
Scarus prasiognathos	0.00 ± 0.00	0.00 ± 0.00	0.00 ± 0.00	0.00 ± 0.00
Scarus viridifucatus	0.00 ± 0.00	0.56 ± 0.37	0.20 ± 0.15	0.00 ± 0.00
Scarus russelii	0.00 ± 0.00	0.00 ± 0.00	0.00 ± 0.00	0.00 ± 0.00
Hipposcarus harid	0.00 ± 0.00	0.11 ± 0.03	2.10 ± 1.17	0.77 ± 0.31
Cetoscarus ocellatus	0.00 ± 0.00	0.00 ±0.00	47.49 ± 10.39	77.03 ± 22.63
Calotomus carolinus	0.00 ± 0.00	0.00 ± 0.00	0.00 ± 0.00	0.00 ± 0.00
Juveniles	0.37 ± 0.19	0.00 ± 0.00	0.00 ± 0.00	0.00 ± 0.00

Supplementary Table 13: Total sediment production rates for Vavvaru parrotfish in the Nearshore – Z3 habitat.

Neashore	Fish Size			
**(g m^{-2} yr^{-1})**	**1 to 15 cm**	**16 to 30 cm**	**31 to 45 cm**	**46 to 60 cm**
Chlorurus sordidus	0.08 ± 0.06	2.86 ± 0.35	0.00 ± 0.00	0.00 ± 0.00
Chlorurus strongylocephalus	0.00 ± 0.00	0.00 ± 0.00	0.00 ±0.00	0.00 ± 0.00
Chlorurus enneacanthus	0.00 ± 0.00	0.00 ± 0.00	0.00 ± 0.00	0.00 ± 0.00
Scarus frenatus	0.00 ± 0.00	0.00 ± 0.00	0.00 ± 0.00	0.00 ± 0.00
Scarus rubroviolaceus	0.00 ± 0.00	0.28 ± 0.18	7.93 ± 2.01	0.05 ± 0.02
Scarus psittacus	0.00 ± 0.00	2.57 ± 0.70	0.00 ± 0.00	0.00 ± 0.00
Scarus niger	0.00 ± 0.00	2.62 ± 0.51	0.00 ± 0.00	0.00 ± 0.00
Scarus tricolor	0.00 ± 0.00	0.00 ± 0.00	0.00 ± 0.00	0.00 ± 0.00
Scarus scaber	0.00 ± 0.00	0.00 ± 0.00	3.00 ± 0.51	0.00 ± 0.00
Scarus prasiognathos	0.00 ± 0.00	0.00 ± 0.00	0.00 ± 0.00	0.00 ± 0.00
Scarus viridifucatus	0.00 ± 0.00	0.00 ± 0.00	0.00 ± 0.00	0.00 ± 0.00
Scarus russelii	0.00 ± 0.00	0.00 ± 0.00	0.00 ± 0.00	0.00 ± 0.00
Hipposcarus harid	0.00 ± 0.00	0.00 ± 0.00	0.17 ± 0.05	0.34 ± 0.10
Cetoscarus ocellatus	0.00 ± 0.00	0.00 ± 0.00	0.00 ± 0.00	0.00 ± 0.00
Calotomus carolinus	0.00 ± 0.00	0.00 ± 0.00	0.00 ± 0.00	0.00 ± 0.00
Juveniles	0.00 ± 0.00	0.00 ± 0.00	0.00 ± 0.00	0.00 ± 0.00

Parrotfish sediment size fractions and composition

All sediment size fraction units are percentages (%)

Supplementary Table 14: Percentage of sediment size fractions produced by different size classes of *Chlorurus sordidus*

Grain Size μm	1 to 15 cm	16 to 30 cm	31 to 45 cm	46 to 60 cm
**32**	4.96 ± 1.02	8.13 ± 1.24	6.02 ± 1.30	N/A
**63**	5.55 ± 0.71	8.30 ± 0.95	6.87 ± 1.11	N/A
**125**	9.81 ± 1.30	14.28 ± 1.11	12.09 ± 0.95	N/A
**250**	18.93 ± 1.91	21.91 ± 1.07	22.36 ± 1.89	N/A
**500**	29.62 ± 1.34	26.71 ± 1.50	30.32 ± 1.70	N/A
**1000**	26.31 ± 2.94	19.40 ± 2.64	21.99 ± 1.81	N/A
**1400**	2.73 ± 1.20	0.90 ± 0.61	0.36 ± 0.23	N/A
**2000**	2.09 ± 2.09	0.38 ± 0.38	0	N/A

Supplementary Table 15: Percentage of sediment size fractions produced by different size classes of *Chlorurus strongylocephalus*

Grain Size μm	1 to 15 cm	16 to 30 cm	31 to 45 cm	46 to 60 cm
**32**	N/A	7.74 ± 1.82	3.18 ± 0.74	6.37 ± 2.40
**63**	N/A	6.99 ± 1.21	4.70 ± 1.04	6.09 ± 1.59
**125**	N/A	12.23 ± 1.57	11.94 ± 1.65	11.37 ± 2.09
**250**	N/A	21.43 ± 1.75	27.95 ± 3.14	21.24 ± 1.75
**500**	N/A	29.10 ± 1.83	32.64 ± 2.72	28.36 ± 2.97
**1000**	N/A	21.72 ± 3.36	18.73 ± 3.00	24.62 ± 4.51
**1400**	N/A	0.48 ± 0.48	0.31 ± 0.31	1.60 ± 0.68
**2000**	N/A	0.31 ± 0.31	0.56 ± 0.56	0.35 ± 0.35

Supplementary Table 16: Percentage of sediment size fractions produced by different size classes of *Scarus frenatus*

Grain Size μm	1 to 15 cm	16 to 30 cm	31 to 45 cm	46 to 60 cm
**32**	8.21 ± 0.92	9.21 ± 1.32	4.71 ± 0.78	4.46 ± 0.85
**63**	11.98 ± 0.53	9.48 ± 1.13	6.26 ± 1.22	6.11 ± 1.14
**125**	20.84 ± 0.89	16.61 ± 1.34	12.84 ± 2.18	12.12 ± 1.16
**250**	26.77 ± 1.70	25.22 ± 1.30	23.97 ± 2.99	24.55 ± 1.03
**500**	20.42 ± 1.07	24.38 ± 1.79	26.26 ± 3.04	31.84 ± 1.46
**1000**	11.79 ± 1.77	14.98 ± 1.47	17.58 ± 1.67	19.66 ± 1.95
**1400**	0	0.06 ± 0.04	7.62 ± 7.62	0.98 ± 0.69
**2000**	0	0.06 ± 0.06	0.76 ± 0.76	0.29 ± 0.29

Supplementary Table 17: Percentage of sediment size fractions produced by different size classes of *Scarus niger*

Grain Size µm	1 to 15 cm	16 to 30 cm	31 to 45 cm	46 to 60 cm
**32**	9.40 ± 2.92	7.96 ± 2.05	7.93 ± 1.77	N/A
**63**	9.90 ± 2.37	7.77 ± 1.38	8.74 ± 1.60	N/A
**125**	17.40 ± 1.55	13.43 ± 1.43	16.60 ± 1.52	N/A
**250**	24.57 ± 1.64	21.18 ± 1.67	28.12 ± 2.17	N/A
**500**	23.85 ± 3.18	25.81 ± 1.93	25.64 ± 1.60	N/A
**1000**	14.00 ± 2.87	21.62 ± 3.09	12.86 ± 4.11	N/A
**1400**	0.35 ± 0.19	1.94 ± 0.76	0.11 ± 0.11	N/A
**2000**	0.53 ± 0.53	0.31 ± 0.21	0	N/A

Supplementary Table 18: Percentage of sediment size fractions produced by different size classes of *Scarus psittacus*

Grain Size µm	1 to 15 cm	16 to 30 cm	31 to 45 cm	46 to 60 cm
**32**	1.99 ± 1.14	9.21 ± 1.09	N/A	N/A
**63**	3.65 ± 1.23	8.93 ± 0.55	N/A	N/A
**125**	8.94 ± 1.80	12.58 ± 2.53	N/A	N/A
**250**	25.44 ± 2.76	23.54 ± 0.26	N/A	N/A
**500**	36.77 ± 2.70	29.78 ± 1.40	N/A	N/A
**1000**	23.21 ± 4.22	15.96 ± 0.32	N/A	N/A
**1400**	0	0	N/A	N/A
**2000**	0	0	N/A	N/A

Supplementary Table 19: Percentage of sediment size fractions produced by different size classes of *Scarus rubroviolaceus*

Grain Size µm	1 to 15 cm	16 to 30 cm	31 to 45 cm	46 to 60 cm
**32**	3.21 ± 1.20	6.36 ± 1.71	2.88 ± 0.51	3.39 ± 0.28
**63**	3.82 ± 0.97	7.29 ± 1.97	3.61 ± 0.56	4.47 ± 0.34
**125**	5.79 ± 1.21	11.96 ± 2.76	8.46 ± 1.46	9.65 ± 0.71
**250**	21.05 ± 3.06	25.23 ± 1.03	21.55 ± 1.98	21.25 ± 1.58
**500**	38.96 ± 2.74	32.48 ± 3.99	33.60 ± 2.82	31.87 ± 0.69
**1000**	25.88 ± 4.11	16.52 ± 2.60	27.84 ± 2.35	26.17 ± 2.09
**1400**	1.30 ± 0.65	0.17 ± 0.11	1.81 ± 1.05	1.85 ± 0.69
**2000**	0	0	0.26 ± 0.26	1.36 ± 0.88

Grain type units are percentages (%)

Supplementary Table 20: Grain types produced by different size classes of *Chlorurus sordidus.* CCA = Crustose Coralline Algae

Grain Type	1 to 15 cm	16 to 30 cm	31 to 45 cm	46 to 60 cm
Coral	91.13	78.74	92.19	N/A
Halimeda spp.	6.73	12.93	3.75	N/A
CCA	0.92	5.46	0.00	N/A
Mollusca	1.22	2.01	2.50	N/A
Foraminifera	0.00	0.00	0.31	N/A
Unidentified	0.00	0.86	1.25	N/A

Supplementary Table 21: Grain types produced by different size classes of *Chlorurus strongylocephalus.* CCA = Crustose Coralline Algae

Grain Type	1 to 15 cm	16 to 30 cm	31 to 45 cm	46 to 60 cm
Coral	N/A	97.83	95.67	93.60
Halimeda spp.	N/A	0.00	1.00	3.66
CCA	N/A	2.17	2.33	2.44
Mollusca	N/A	0.00	0.00	0.00
Foraminifera	N/A	0.00	0.00	0.30
Unidentified	N/A	0.00	1.00	0.00

Supplementary Table 22: Grain types produced by different size classes of *Scarus frenatus..* CCA = Crustose Coralline Algae

Grain Type	1 to 15 cm	16 to 30 cm	31 to 45 cm	46 to 60 cm
Coral	95.51	97.85	87.57	96.71
Halimeda spp.	1.40	1.53	1.16	0.00
CCA	2.25	0.31	4.34	1.52
Mollusca	0.56	0.00	4.91	0.51
Foraminifera	0.00	0.31	2.02	1.27
Unidentified	0.28	0.00	0.00	0.00

Supplementary Table 23: Grain types produced by different size classes of *Scarus niger..* CCA = Crustose Coralline Algae

Grain Type	1 to 15 cm	16 to 30 cm	31 to 45 cm	46 to 60 cm
Coral	98.60	97.39	95.02	N/A
Halimeda spp.	0.23	0.65	0.93	N/A
CCA	0.47	0.98	2.18	N/A
Mollusca	0.23	0.65	1.25	N/A
Foraminifera	0.47	0.33	0.31	N/A
Unidentified	0.00	0.00	0.31	N/A

Supplementary Table 24: Grain types produced by different size classes of *Scarus psittacus..* CCA = Crustose Coralline Algae

**Grain Type**	**1 to 15 cm**	**16 to 30 cm**	**31 to 45 cm**	**46 to 60 cm**
Coral	96.60	92.05	N/A	N/A
Halimeda spp.	0.85	0.66	N/A	N/A
CCA	2.55	4.97	N/A	N/A
Mollusca	0.00	1.66	N/A	N/A
Foraminifera	0.00	0.66	N/A	N/A
Unidentified	0.00	0.00	N/A	N/A

Supplementary Table 25: Grain types produced by different size classes of *Scarus rubroviolaceus..* CCA = Crustose Coralline Algae

**Grain Type**	**1 to 15 cm**	**16 to 30 cm**	**31 to 45 cm**	**46 to 60 cm**
Coral	100.00	100.00	92.90	97.78
Halimeda spp.	0.00	0.00	2.16	0.28
CCA	0.00	0.00	3.40	1.39
Mollusca	0.00	0.00	0.93	0.00
Foraminifera	0.00	0.00	0.31	0.28
Unidentified	0.00	0.00	0.31	0.28

Habitat Sediment Data

Supplementary Table 26: Sediment load within the epilithic algal matrix in each Vavvaru habitat

Habitat	Sediment Load (g cm^{-3} ± SE)
Hardground	0.008 ± 0.001
Rubble	0.01 ± 0.003
Porites bommie	0.01 ± 0.003
NE reef	0.019 ± 0.013
SE patches	0.024 ± 0.009
Nearshore lagoon	0.021 ± 0.005

Supplementary Table 27: Grainsize distribution of benthic sediments in Vavvaru habitats (units = %)

Habitat	Hardground	Rubble	Porites	NE	SE	Nearshore
**32**	10.51 ± 2.42	9.20 ± 1.96	4.32 ± 1.38	8.26 ± 2.10	7.26 ± 2.32	4.39 ± 1.07
**63**	12.91 ± 1.79	10.00 ± 1.41	5.60 ± 1.05	12.76 ± 3.29	8.06 ± 2.41	6.73 ± 0.65
**125**	17.10 ± 2.01	15.13 ± 1.13	11.57 ± 0.44	28.39 ± 3.36	18.25 ± 1.96	21.69 ± 2.08
**250**	18.91 ± 3.03	27.97 ± 1.63	29.40 ± 2.67	30.83 ± 3.25	33.91 ± 3.38	36.44 ± 1.67
**500**	18.40 ± 2.28	27.12 ± 2.24	33.90 ± 0.91	14.90 ± 4.27	24.82 ± 3.54	22.23 ± 3.41
**1000**	18.98 ± 5.12	10.46 ± 1.73	14.58 ± 1.91	4.40 ± 1.19	6.55 ± 0.15	6.64 ± 1.72
**1400**	0.66 ± 0.66	0.08 ± 0.08	0.21 ± 0.09	0.06 ± 0.06	0.14 ± 0.07	0.51 ± 0.43
**2000**	0.82 ± 0.82	0.03 ± 0.03	0.41 ± 0.33	0.40 ± 0.24	0.16 ± 0.16	1.38 ± 1.23
**2800**	1.11 ± 1.11	0.00 ± 0.00	0.00 ± 0.00	0.00 ± 0.00	0.83 ± 0.42	0.00 ± 0.00

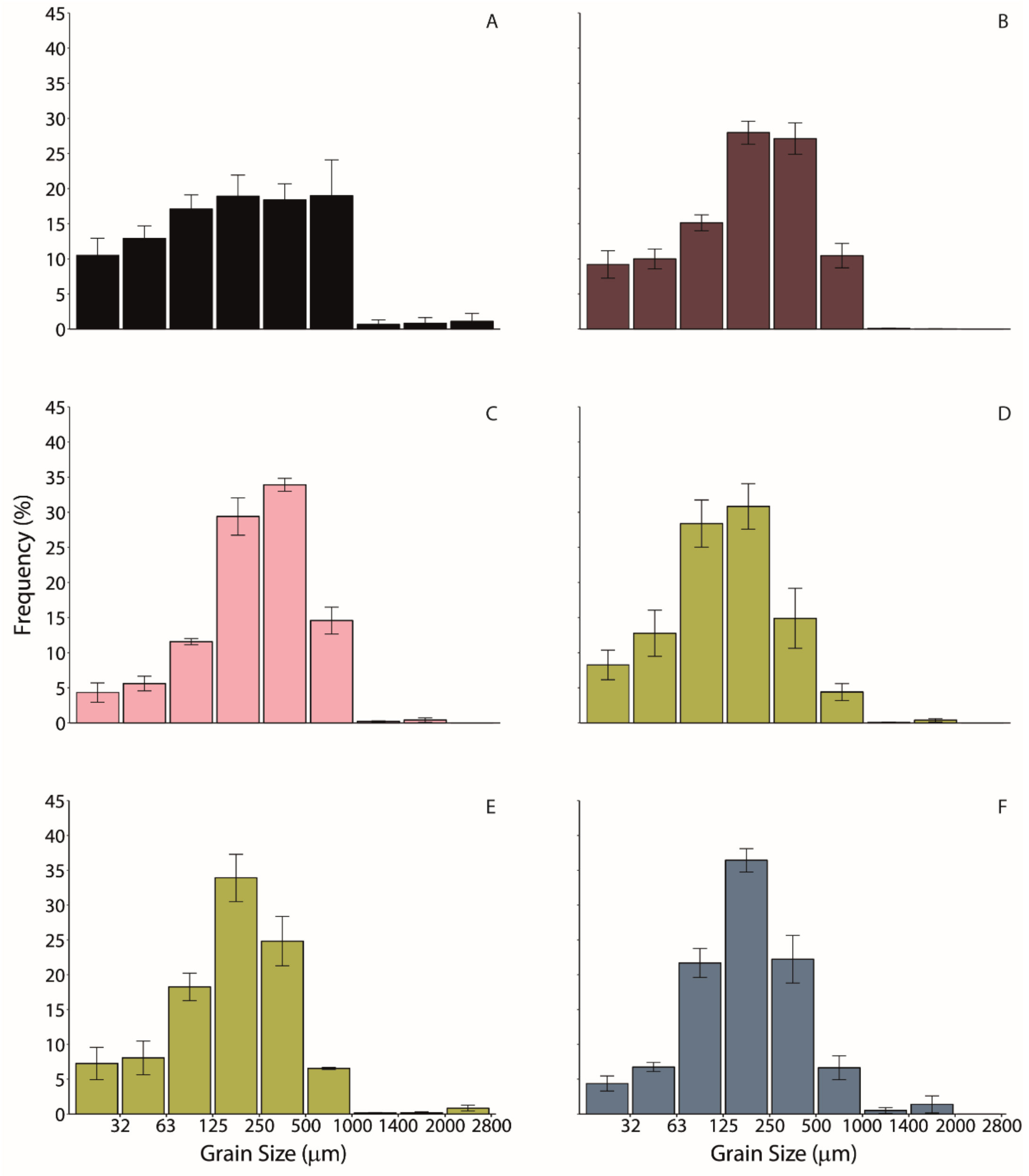

Fig S3: Grainsize distribution of benthic sediments in Vavvaru habitats. A = Hardground, B = Rubble, C = Porites bommie, D = NE reef, E = SE reef and F = Nearshore lagoon

Supplementary Table 28: Percent (%) contribution of grain types found in EAM sediments in each Vavvaru habitat. CCA = Crustose Coralline Algae

Grain Type	Hardground	Rubble	Porites	NE	SE	Nearshore
Coral	97.94	94.06	98.31	96.78	97.10	97.55
Halimeda	0.88	1.65	0.56	0.80	1.61	1.22
CCA	0.00	1.65	0.56	0.00	0.97	0.92
Mollusca	0.88	1.98	0.28	1.88	0.00	0.00
Foraminifera	0.29	0.00	0.28	0.27	0.32	0.31
Unidentified	0.00	0.00	0.00	0.27	0.00	0.00
